# Hydrologic Variability Affects Invertebrate Grazing on Phototrophic Biofilms in Stream Microcosms

**DOI:** 10.1371/journal.pone.0060629

**Published:** 2013-04-16

**Authors:** Serena Ceola, Iris Hödl, Martina Adlboller, Gabriel Singer, Enrico Bertuzzo, Lorenzo Mari, Gianluca Botter, Johann Waringer, Tom J. Battin, Andrea Rinaldo

**Affiliations:** 1 Laboratory of Ecohydrology (ECHO/IEE/ENAC), École Polytechnique Fédérale de Lausanne, Lausanne, Switzerland; 2 Dipartimento di Ingegneria Civile, Chimica, Ambientale e dei Materiali, Università di Bologna, Bologna, Italy; 3 Department of Limnology, University of Vienna, Vienna, Austria; 4 Dipartimento di Elettronica, Informazione e Bioingegneria, Politecnico di Milano, Milano, Italy; 5 Dipartimento di Ingegneria Civile, Edile ed Ambientale, Università degli Studi di Padova, Padova, Italy; 6 WasserCluster Lunz, Interuniversity Center for Aquatic Ecosystem Research, Lunz am See, Austria; Auburn University, United States of America

## Abstract

The temporal variability of streamflow is known to be a key feature structuring and controlling fluvial ecological communities and ecosystem processes. Although alterations of streamflow regime due to habitat fragmentation or other anthropogenic factors are ubiquitous, a quantitative understanding of their implications on ecosystem structure and function is far from complete. Here, by experimenting with two contrasting flow regimes in stream microcosms, we provide a novel mechanistic explanation for how fluctuating flow regimes may affect grazing of phototrophic biofilms (i.e., periphyton) by an invertebrate species (*Ecdyonurus* sp.). In both flow regimes light availability was manipulated as a control on autotroph biofilm productivity and grazer activity, thereby allowing the test of flow regime effects across various ratios of biofilm biomass to grazing activity. Average grazing rates were significantly enhanced under variable flow conditions and this effect was highest at intermediate light availability. Our results suggest that stochastic flow regimes, characterised by suitable fluctuations and temporal persistence, may offer increased windows of opportunity for grazing under favourable shear stress conditions. This bears important implications for the development of comprehensive schemes for water resources management and for the understanding of trophic carbon transfer in stream food webs.

## Introduction

The study of flow regime as the master variable controlling fluvial ecological and geomorphological processes, from river network evolution [1] to biodiversity distribution and benthic biota interactions [2]–[6], lies at the heart of ecohydrology. Streamflow is an interactive byproduct of rainfall, climate, land use and geomorphology [1], [7]–[9]. It controls life in streams and rivers [2], [10], [11], sustaining and regulating their ecosystem integrity [12]–[14]. The flow environment, in terms of discharge, water depth, flow velocity and bottom shear stress, shapes, for instance, the physical structure and community composition of benthic biofilms, which constitute the trophic basis for numerous benthic organisms [15]–[17]. Streamflow may also control the dispersal, distribution and foraging behaviour of stream invertebrates – a focal research point in stream ecology over the last decades [18]–[24]. Integrative approaches, including trophic interactions, are necessary to understand the effects of flow regime on benthic life [3]. However, few theoretical or experimental studies have explicitly considered the effects of flow variability on ecological processes like: resource acquisition [2], [3], [25]–[29]; community organisation as expressed for example by food chain length [30], [31]; habitat suitability for algae or invertebrates [23], [32]; and thermopeaking waves due to release from reservoirs [33], [34].

Another fundamental control on stream ecosystem structure and function is light availability [11], which typically changes along the fluvial continuum [35] and may limit primary productivity, consequently affecting the stream food web structure and energy flow [36]. Several studies have analysed the effects of light availability on algal communities and on bottom-up effects on macroinvertebrates [16], [36], [37], but the coupled effects of flow and light regimes on biofilm grazing, and hence on the trophic transfer of carbon, remain poorly understood. Unraveling such underlying mechanisms is relevant for several reasons. Increasing perturbation of flow regimes and riparian deforestation altering the light regime in headwaters may affect trophic interactions, which greatly contribute to ecosystem functioning.

**Figure 1:**
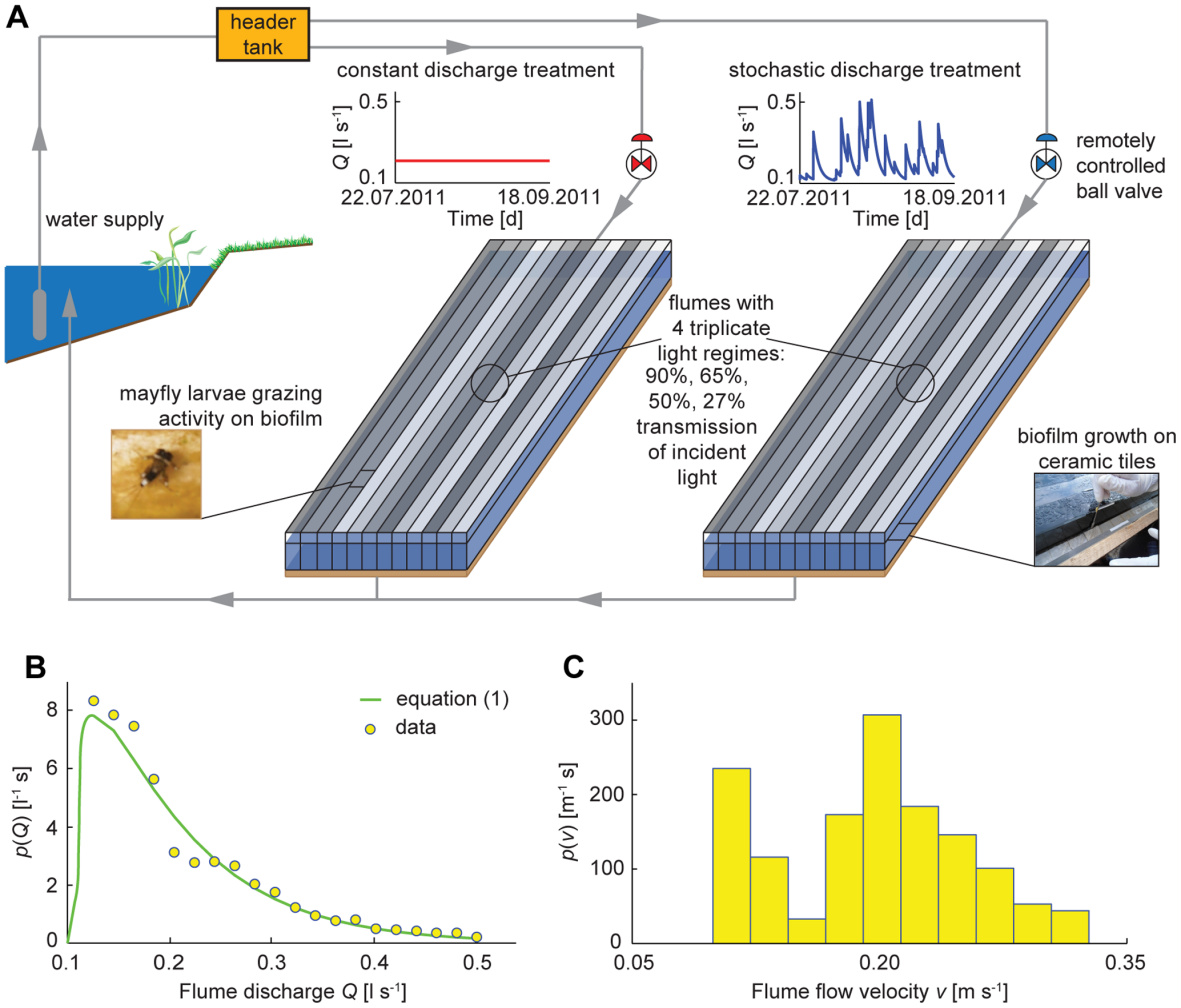
Experimental setup. (a) Schematic representation of the experimental facilities, with details on discharge and light treatments, biofilm growth on tiles and mayfly larvae grazing activity. (b) Probability distribution function of flume discharge 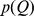: dots represent the experimental distribution, the green solid line shows the imposed distribution. (c) Probability distribution function of flume flow velocity, 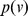, derived from measured data.

To test possible effects of the temporal variation of streamflow and light availability on biofilm-grazer trophic interactions, we experimented with microcosms in which we grew benthic phototrophic biofilms from raw water and generated a time-variable streamflow sequence, obtained from a probability distribution derived analytically from general hydrologic assumptions (Figure 1). Additional control flumes had a constant flow regime, equivalent to the average discharge of the stochastic flow treatments. Four levels of light availability were introduced as triplicates in each flow treatment. A larval mayfly (*Ecdyonurus* sp.) was selected as model grazer that typically occurs in pre-alpine streams, including our study stream Oberer Seebach (OSB).

## Materials and Methods

### Experimental Setup

The experiment was conducted at the WasserCluster Lunz, in Lunz am See (Austria, 47.86° N, 15.05° E), from July 22*^nd^* to September 18*^th^* 2011. The design consisted of 24 flumes (12 for each discharge treatment) 3 m long, 0.1 m deep, 0.05 m wide, with a slope of 0.003, that were operated in once-through flow mode. Flumes were made from two Plexiglas slabs with internal partitions. Commercially available low-porosity unglazed ceramic tiles (Villeroy & Boch, Germany) with an approximate size of 5×5 cm paved each flume and constituted a suitable substratum for biofilm growth and grazing activity of mayfly larvae [38]. Water was supplied through a submerged pump, with temperature between 10.5°C and 13.9°C. A header tank received the pumped water which flowed into two pipes (one for each discharge treatment) at the bottom of the tank, and then entered two smaller tanks that supplied the flumes (Figure 1A and Figure S6, S7, S8, S9 in Supporting Information). The designed setup ensured that all flumes belonging to the same discharge treatment experienced identical hydraulic conditions, as the flume water level equalled the water level of the small tank. To regulate and record the temporal sequence of volumetric flow rate, i.e., discharge, a valve and a propeller flow meter were placed in each of the two supply pipes. The various components of the setup were covered to avoid wind-blown inputs (e.g., rain, leaves, insects). At the flume inlet, 1.75 m downstream, and at the flume outlet three nets made of stainless steel wire were placed to regulate flow, enhance uniform flow conditions, sustain water level and confine mayfly larvae. The flume outlet was open and water freely flowed into a small channel. For full technical details see Text S1 in Supporting Information.

### Discharge Treatments

A stochastic and a constant discharge treatment were performed (Figure 1A). Temporal changes in flow were implemented through a numerical simulation (i.e., Monte Carlo realisation) of a stochastic process which is capable of reproducing the relevant streamflow dynamics observed in world-wide river catchments. More specifically, daily streamflow dynamics are assumed to result from the superposition of a sequence of water impulses triggered by precipitation. The sequence of runoff-producing rainfall events is a suitable subset of all rainfall events, filtered by singling out those bringing enough water to fill the water deficit created by plant transpiration in the root zones of the entire catchment, and drive the soil water content in this region above the retention point. Therefore, such pulses determine an excess of water in the root zones, which is eliminated through the hydrologic response of the catchment – the streamflow, 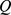 [L^3^ T^−1^]. Therefore, the temporal sequence of discharges typical of unregulated streams is characterised by sudden increments due to rainfall events producing streamflow, followed by slower recession phases determined by the distribution of times needed by the hydrologic signal to propagate to the outlet through the whole catchment (e.g., Figure 1A, for the stochastic discharge treatment). From a mathematical perspective, daily rainfall events represent a stochastic process usually modelled as a marked Poisson process [39], [40] characterised by the frequency of rainfall interarrivals, 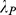 [T^−1^], and by exponentially distributed precipitation depths with mean 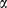 [L]. Daily soil moisture dynamics in the near-surface soil layer are mainly controlled by evapotranspiration and deep percolation processes that contribute to streamflow production [40]. Daily rainfall events producing streamflow can thus be modelled as a marked Poisson process characterised by the same mean rainfall depth as rainfall events and a lower frequency [40], 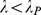 [T^−1^]. In practice, this means that the streamflow-producing rainfall events have an instantaneous duration triggering a sequence of localised jumps in the streamflow that are then released from the soil to the channel network following an exponential response function with mean response time 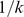 [T], proportional to the catchment area 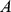 [L^2^]. A stochastic dynamic reproducing these two fundamental processes [8], which proved to be able to remarkably well reproduce the observed behaviour of many catchments throughout the world characterised by different climatic and morphologic attributes [41], [42], reads 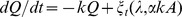, where 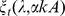 is a marked Poisson noise describing the random arrivals of exponentially distributed instantaneous streamflow jumps triggered by rainfall events. The parameter 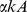 [L^3^ T^−1^] represents the mean discharge jump [8]. The resulting probability distribution function of streamflows can be expressed as [8]:
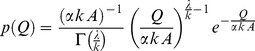
(1)where 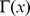 is the complete Gamma function of argument 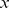.

In order to reproduce streamflow dynamics typical of unaltered streams in our experimental flumes, a realisation of the aforementioned stochastic process was simulated. Given that the experiment was conducted in a pre-alpine area (Oberer Seebach, Austria), a time-varying discharge treatment that reflects the typical characteristics of pre-alpine streams was performed. From previous analyses in several pre-alpine catchments [42], where parameter values had been estimated directly from rainfall and discharge data, it was found that typical values of 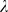 during the summer period were in the range from 0.4 to 0.8 d^−1^. For our experiment we selected 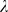 = 0.6 d^−1^. The parameter 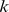 was set equal to 0.5 d^−1^, which is appropriate for headwater catchments whose size is of the order of 1 to a few km^−2^. The ratio 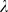/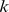 determines the shape of the streamflow distribution [8], [42]. In this case the distribution is hump-shaped like those typically characterising perennial streams in pre-alpine catchments during summer. Finally, the product 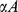 [L^3^] determines the magnitude of streamflows and was chosen so as to produce a range of discharges that suited the minimum and maximum depths allowed by the size of the experimental flumes. A reasonable range of hydraulic conditions (velocity, shear stress) was thus generated. As a result, the microcosms experienced a rescaled range of discharges, whose variability (coefficient of variation, 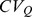) matches the variability characteristically found for perennial headwater pre-alpine catchments [42] (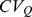 = 0.5–1). A constant discharge treatment, with a discharge equal to the average of the stochastic sequence, was performed as a control. To implement and control the temporal sequence of the stochastic discharge regime, a computer-controlled system was developed using National Instruments LabVIEW ™ software, which regulated a calibrated electric ball valve. A manual ball valve was used to set the constant discharge regime. An analog input module (NI 9203), based on a current signal of 0–20 mA, was used to register the discharge values measured by the two flow meters, while an analog output module (NI 9263), producing a voltage signal of 0–10 V, was used to command the opening temporal sequence of the electric valve. These modules were placed into a chassis (NI cDAQ-9174), connected to a computer via a USB interface.

### Hydraulic Properties

Temporal changes of discharge, 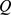 [L^3^ T^−1^], are reflected in changes of other hydraulic characteristics, such as water depth, 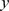, flow velocity, 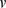, bottom shear stress, 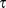, and shear velocity, 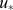. Flow velocity, 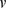 [L T^−1^], is defined as: 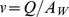 where 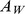 [L^2^] is the flume wetted cross section (in the case at hand 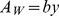, where 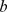 [L] and 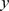 [L] represent channel width and depth). The cross-section average bottom shear stress, 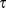 [M L^−1^ T^−2^] exerted on the wetted perimeter can be expressed in the uniform flow conditions maintained here as 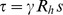 where 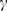 [M L^−2^ T^−2^] is the specific weight of water, 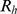 [L] is the hydraulic radius (the ratio of wetted area and perimeter, here 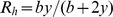) and 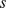 is the flume slope. Shear velocity, 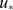 [L T^−1^], represents the friction velocity at the bottom of a channel, and, under uniform flow conditions, is 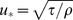 where 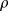 [M L^−3^] is water density. Hydraulic relationships derived from flume discharge and water depth measurements are reported in Table 1.

**Table 1:** Hydraulic relationships for the stochastic discharge treatment.

Relation	Exponent	Value
*y* ∝ *Q^φ^*	*φ*	0.57
*v* ∝ *Q^β^*	*β*	0.43
*τ* ∝ *Q^δ^*	*δ*	0.24
u_*_ ∝ *Q^η^*	*η*	0.12

### Probability Distribution Function of Bed Shear Stress

Given the one-to-one relation between streamflow, 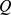, and shear stress, 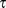 (i.e., 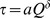, where 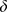 = 0.24), postulated by the uniform flow conditions maintained experimentally, the analytical expression of the probability distribution function of shear stress can be obtained as a derived distribution from the streamflow probability distribution (equation (1)), as:
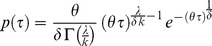
(2)where 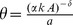 is the inverse of bottom shear stress corresponding to a discharge condition equal to the mean streamflow increment due to incoming streamflow-producing rainfall events, 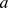 and 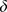 are the parameters defining the relation between 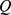 and 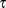, and 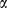, 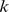, and 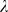 are the parameters of the streamflow probability distribution function (equation (1)).

### Light Treatments

Lighting filters, providing different light intensities without changing the spectral distribution, were selected to reproduce distinct light conditions. In particular, neutral density grey filters 226, 298, 209, 210 were used to create respectively 90%, 65%, 50%, and 27% transmission of incident light (as PAR). For each discharge regime, three replicates of each PAR treatment were set up to provide a sufficiently large sample size. Foils were placed randomly on the 12 flumes (Figure S10 in Supporting Information). The positioning scheme of PAR treatments was the same for both discharge treatments.

### Invertebrate Grazer Species

Mayflies (*Ecdyonurus* sp.) at a larval stage (summer and fall generations) were used during the experiment. Invertebrates were collected from the Oberer Seebach (OSB), a pre-alpine third order stream in Lunz am See, Austria. The OSB and its tributaries have been the focus of macrozoobenthos research over many years (Table S1 in Text S1 in Supporting Information). Four *Ecdyonurus* species are known from these streams: *E. dispar* (Curtis, 1834), *E. venosus* (Fabricius, 1775), *E. helveticus* (Eaton, 1885) and *E. picteti* (Meyer-Dür, 1864). More than 95% of the larvae belonged to late instars of *E. helveticus*, with the others distributed over the remaining three species. All four species have identical feeding habits and are assigned to the same functional feeding groups (FFG): 50% grazer/scraper and 50% detritivore/gatherer/collector [43]. Based on a recent survey in OSB, the typical composition of FFG is as follows: shredders (16%), detritivores/gatherers/collectors (40%), grazers/scrapers (26%), filtering collectors (3%), predators (14%) and parasites (<1%). The grazers/scrapers include on average the following taxa: Gastropoda (<1%), Amphipoda (<1%), Ephemeroptera (18%), Plecoptera (38%), Coleoptera (4%), Trichoptera (1%) and Diptera (37%). The Ephemeroptera consist of the following genera: *Baetis* (42%), *Ephemerella* (10%), *Ecdyonurus* (45%) and *Epeorus* (3%). This detailed information supports the choice of *Ecdyonurus* as a focal and model grazer for our experiments. Animals were collected in the OSB, from three weeks to two days before their inclusion in the flumes (i.e., September 2^nd^). Mayfly larvae were kept in buckets of stream water, aerated, and maintained in a climate chamber at a temperature of 10°C (nearly the same temperature of OSB stream-water and the water in the flumes). Eight *Ecdyonurus* larvae were inserted in the upstream segment of each flume at the onset of the grazing period. Care was taken to randomly choose the invertebrates from a bucket and avoid the introduction of any sampling effect into the experimental design. In order to have a constant grazing pressure, alive and dead grazers were counted every night, and dead or missing grazers were replaced. At the end of the experiment, all *Ecdyonurus* larvae were removed in order to determine their dry mass and to analyse their gut contents.

### Experimental Procedure

Daily analyses consisted of measurements of water level in each flume and water temperature both in the flumes and in the header tank. Discharge was measured continuously by the flow meters. To measure biofilm biomass in terms of total organic matter (OM) and algal biomass, expressed as ash-free dry mass [mg cm^−2^] and chlorophyll-*a* (Chl-*a*) [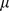
g cm^−2^], respectively, tiles were sampled in intervals of two to seven days. During the initial experimental phase, in which grazers were excluded (from July 22^nd^ to September 1^st^), one tile from each flume was sampled in intervals of two to seven days. The tiles were selected in the final part of the flume, moving upstream and avoiding the last 25 cm, in which uniform hydraulic conditions were not well established. A fresh, uncolonised tile was used for replacement at the sampled position. During the grazed phase (from September 2^nd^ to September 18^th^), three tiles from the upstream part of each flume were sampled in intervals of four days. For each sampling day, one tile from the upper, intermediate and lower part of the upstream flume segment were removed, avoiding the first 35 cm and the last 25 cm, which possibly experienced non-uniform flow conditions. The sampled tiles were replaced by unsampled but colonised tiles from the downstream sector, and the latter were replaced by fresh, uncolonised tiles. See Text S1 in the Supporting Information for more details.

### Biofilm-grazer Dynamics

**Figure 2:**
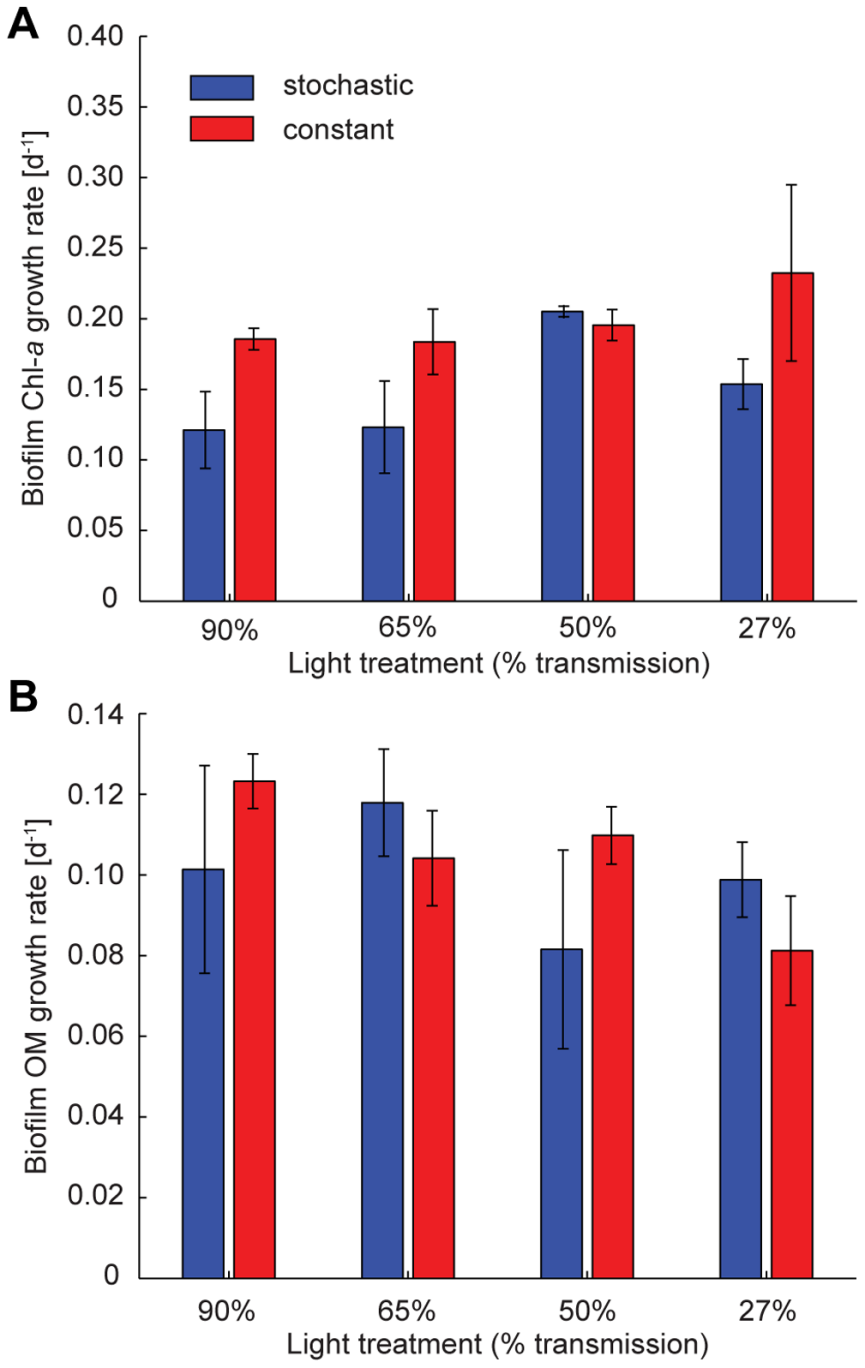
Biofilm growth rate. (a) Biofilm Chl-*a* growth rate [d^−1^] for each discharge and light treatment (mean ± SD). Two-way ANOVA: discharge F_1,16_ = 5.58, P = 0.031; light F_3,16_ = 1.5, P = 0.252; discharge × light F_3,16_ = 0.93, P = 0.451. (b) Biofilm OM growth rate [d^−1^] for each discharge and light treatment (mean ± SD). Two-way ANOVA: discharge F_1,16_ = 0.18, P = 0.676; light F_3,16_ = 1.01, P = 0.413; discharge × light F_3,16_ = 1.15, P = 0.358. Blue and red bars refer to stochastic and constant discharge treatments, respectively.

Biofilm-grazer interactions observed in this experiment can be described mathematically by two equations, namely: i) biofilm dynamics for ungrazed conditions, 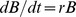 (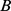 is the biofilm biomass, expressed as OM [mg cm^−2^] or Chl-*a* [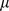
g cm^−2^], 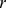 is the net biofilm growth rate [d^−1^]); and ii) biofilm dynamics for grazed conditions 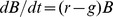 (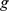 is the grazing rate [d^−1^]). In particular, biofilm growth rate during the ungrazed phase (Figure 2) and *Ecdyonurus* grazing rate during the grazed phase (Figure 5C and Figure S4 in Supporting Information) were evaluated. During the initial ungrazed conditions, where low biofilm density and negligible losses of biomass due to hydraulic stress or cell death are observed, biofilm growth can be described by a simple exponential growth model. Thus temporal biofilm growth can be expressed as 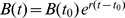 where 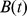 is the biofilm biomass at time 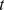, and 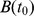 is the initial biofilm biomass. A logarithmic fit of the measured ungrazed biomass for each sampling day was performed (measured biomass value from August 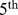 to August 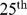, average biomass of triplicate measurements on September 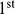). On a semi-log plot (log 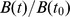 vs 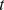) the growth rate is simply the slope of the best fit interpolant (Figure S11 in Supporting Information). The overall grazing rate exerted by the *Ecdyonurus* specimens located in the flumes was computed from the total fraction of biofilm removed by grazing within a certain time interval. Following the model formulation, a logarithmic fit of the measured biomass under ungrazed and grazed conditions was performed on data from the grazed phase in order to get 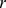 and 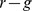, respectively. On a semi-log plot, the grazing rate is the difference of the slopes of the two best fit interpolants (Figure S12 in Supporting Information).

### Autotrophic Community Composition of Benthic Biofilms

The autotrophic community composition (ACC) of benthic biofilms was determined by identification of algal cells in 8 representative samples collected at the onset of the grazing phase from all flow and light treatments. Biofilm samples were scraped from 5.8 cm^2^ (i.e., a quarter of a ceramic tile) and stored in 3.6% formaldehyde. In Utermöhl counting chambers [44] algal cells were identified from a cell suspension from 1∶100 up to 1∶500 depending on the light treatment. For every sample at least 5 Utermöhl chambers were counted, adding up to at least 3,000 identified cells corresponding to 0.6–0.98 mm^2^ of area covered with biofilm. Overall 68 algal taxa were microscopically differentiated at the genus level and counted.

### Statistical Analysis

To test the hypothesis that biofilm and grazer dynamics are controlled by the experimental environmental conditions, an analysis of variance (ANOVA) was performed. In particular the effects of discharge treatment (i.e., stochastic vs constant) and light regime (i.e., 90%, 65%, 50% and 27% transmission of incident PAR) on biofilm growth rate, biofilm biomass before grazer inclusion, Autotrophic Index (AI, i.e., the ratio of biofilm OM to Chl-*a* [45]) and *Ecdyonurus* larvae grazing rate were investigated. The effects of single factors and interactions among them were examined. All tests were considered significant if the *p*-value was less than 0.05; confidence intervals are given at 95%.

In addition, a canonical correlative approach (see Text S1 in Supporting Information) was used to test if the effects of flow and light on grazing rates were mediated by changes in the ACC of benthic biofilms. ACC was also analysed using non-metric multidimensional scaling (NMDS) based on a Bray-Curtis dissimilarity matrix computed from relative algae abundances [46].

### Grazer-induced Organic Carbon Flux from Biofilms

Organic carbon (OC) fluxes derived from phototrophic biofilms and induced by mayfly grazers were estimated from measurements of OM removed by grazing. At each sampling day, the removed OM [mg cm^−2^] was calculated as the difference between the OM under ungrazed and grazed conditions, and the average daily removed biomass [mg cm^−2^ d^−1^] was consequently derived as the ratio of removed biomass to the time interval from grazers’ inclusion. Given that on a conservative basis OC is nearly 45% of OM, the average daily organic carbon flux [mg C cm^−2^ d^−1^] was estimated as a fraction of removed OM (Table 2). Based on *Ecdyonurus* density (i.e., 8 individuals per flume), the average organic carbon flux for each mayfly larvae [mg C d^−1^ individual^−1^] was finally determined. It is important to note that the herein determined removed OM includes OM ingested by mayflies and OM entering the water column, thus experiencing export from the system due to bioturbation [15], [47]. Grazed biofilms have been reported to have increased net productivity due to removal of senescent biofilm biomass and maintenance of biofilm in a young and productive growth stage by “gardening” grazers [15], [48]–[50]. The estimates of OC flux provided here must therefore be considered conservative, i.e., real fluxes may as well be higher, as the simple computation of removed OM by differencing does not account for a potentially increased productivity of grazed biofilms.

**Table 2:** Organic carbon flux [mg C d^−1^ individual^−1^] for each discharge (S for stochastic, C for constant) and light treatment (mean ± SD).

% transmission	S	C
90%	0.46±0.39	0.29±0.19
65%	0.72±0.99	0.47±0.69
50%	1.77±1.35	0.36±0.20
27%	0.79±0.68	1.13±1.27
all light	0.94±1.03	0.56±0.78

## Results

### Benthic Biofilm Growth

Our experimental flumes were characterised by a physical environment that was comparable to shallow streams typical of pre-alpine catchments. Flumes under the stochastic flow treatment where characterised by discharge, 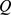, flow velocity, 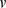 and average bottom shear stress, 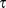, ranging from 0.12 to 0.51 l s^−1^, 0.09 to 0.33 m s^−1^ and 0.26 to 0.50 N m^−2^, respectively. In the constant flow regime, discharge, equivalent to the average value of the stochastic sequence, was set equal to (±standard deviation) 0.21±0.01 l s^−1^, while flow velocity was 0.228±0.001 m s^−1^ and respective bottom shear stress was 0.428±0.001 N m^−2^. Shading flumes yielded average (±standard deviation) daily maximal intensities of PAR of 1130±220 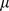
E m^−2^ s^−1^, 864±195 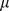
E m^−2^ s^−1^, 625±141 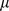
E m^−2^ s^−1^ and 359±81 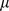
E m^−2^ s^−1^ in the respective light treatments. These averages are within the range of maximum daily PAR values (131 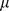
E m^−2^ s^−1^ to 1753 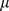
E m^−2^ s^−1^, 1142±384 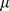
E m^−2^ s^−1^) as measured during the experimental period in OSB.

**Figure 3:**
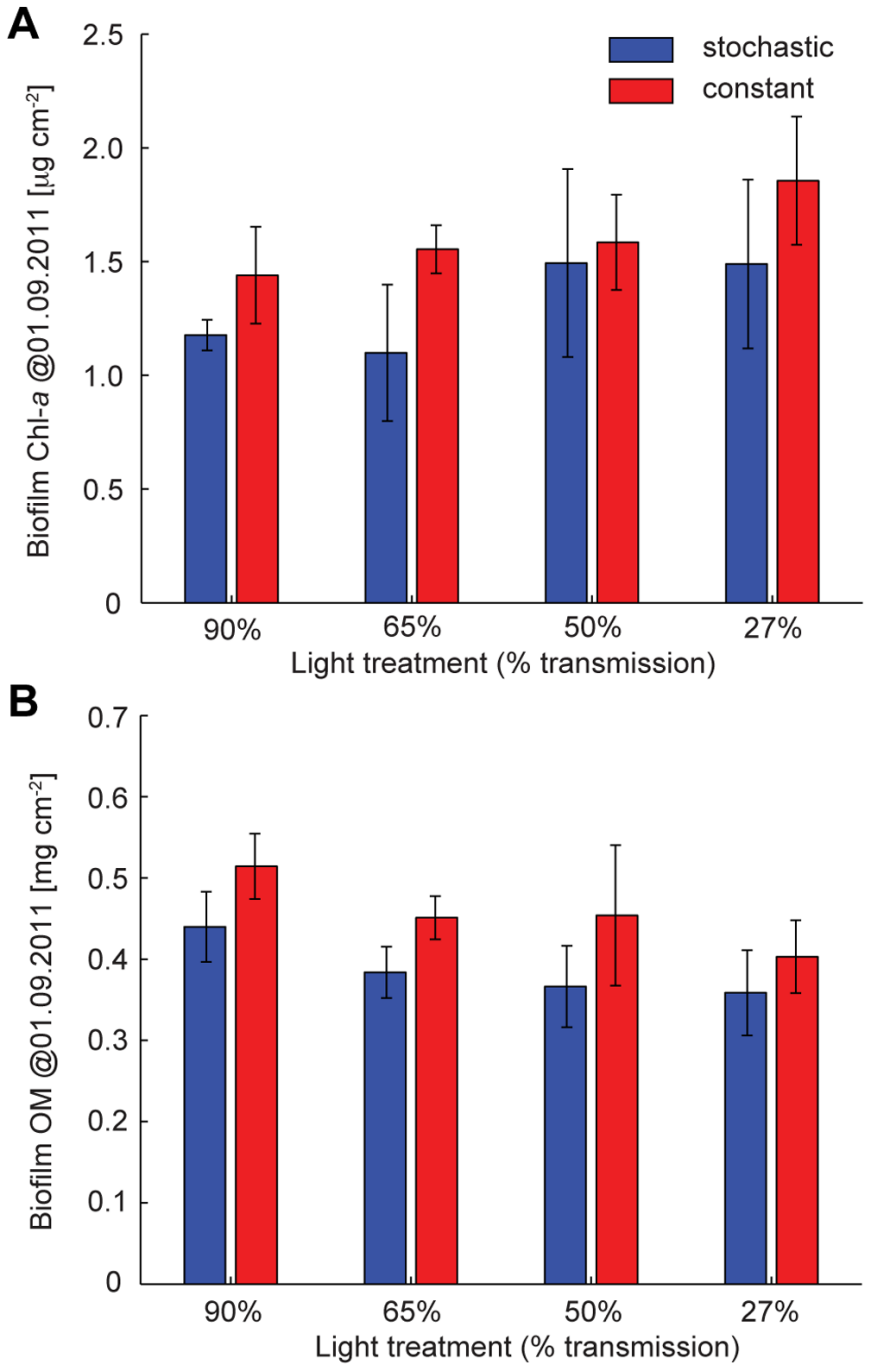
Biofilm biomass before grazers’ inclusion. (a) Biofilm Chl-*a* [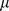
g cm^−2^] for each discharge and light treatment (mean ± SD). Two-way ANOVA: discharge F_1,16_ = 7.12, P = 0.017; light F_3,16_ = 2.53, P = 0.094; discharge x light F_3,16_ = 0.5, P = 0.685. (b) Biofilm OM [mg cm^−2^] for each discharge and light treatment (mean ± SD). Two-way ANOVA: discharge F_1,16_ = 11.25, P = 0.004; light F_3,16_ = 3.92, P = 0.028; discharge×light F_3,16_ = 0.2, P = 0.897. Blue and red bars refer to stochastic and constant discharge treatments, respectively.

**Figure 4:**
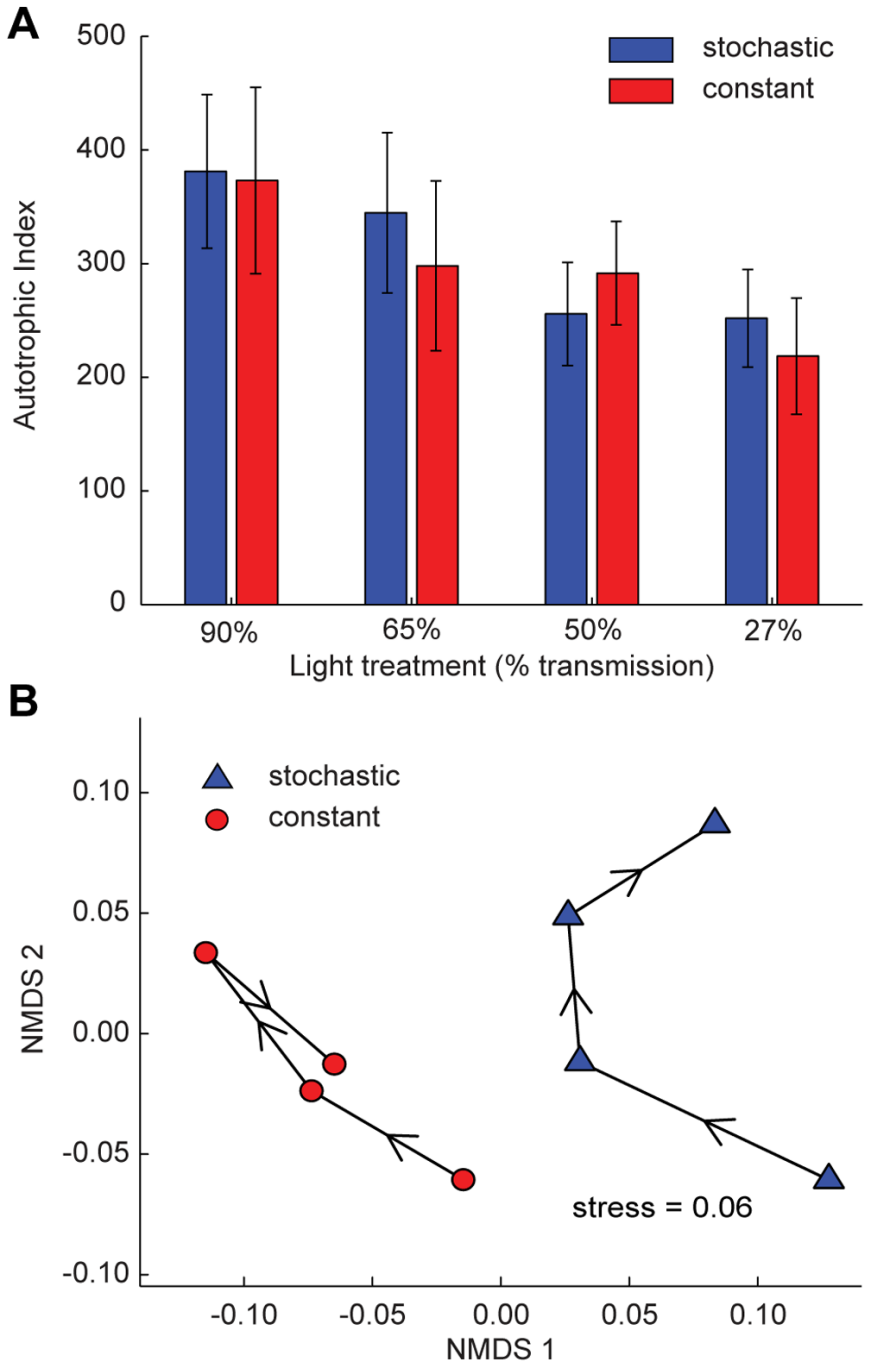
Biofilm biomass analysis. (a) Autotrophic Index before grazers’ inclusion for each discharge and light treatment (mean ± SD). Two-way ANOVA: discharge F_1,16_ = 0.64, P = 0.437; light F_3,16_ = 12.96, P<0.001; discharge x light F_3,16_ = 1.08, P = 0.384. Blue and red bars refer to stochastic and constant discharge treatments, respectively. (b) Non-metric multidimensional scaling based on a Bray-Curtis dissimilarity matrix computed from relative abundances of 68 algal taxa identified from biofilms. Blue triangles and red circles refer to stochastic and constant flow regimes, respectively; arrows indicate the decreasing light gradient created by neutral density grey filters. Note that flow and light cause independent shifts of autotrophic community composition along separate ordination axes, which are, however, similar in magnitude.

In these flumes, phototrophic biofilms grew from raw water in the absence of invertebrate grazers. During this initial phase of the experiment, biofilm growth rate, based on Chl-*a*, was significantly affected by the flow regime but not by the light treatment (Figure 2A and Figure S1 in Supporting Information). Biofilm biomass as bulk organic matter (OM) and Chl-*a* revealed a parallel pattern with significantly lower biomass in the stochastic than in the constant flow regime (Figure 3 and Figure S2 in Supporting Information). In both flow regimes, biofilm OM decreased significantly with decreasing PAR availability, while Chl-*a* was highest in the darkest light treatment (27% transmission). This resulted in a significant decrease of the Autotrophic Index (AI) with decreasing light intensity, while AI remained unaffected by the flow regime (Figure 4A and Table S2 in Text S1 in Supporting Information). Elevated Chl-*a* per unit biomass likely reflects a physiological response of algae to cope with reduced PAR availability [37], whereas photo-inhibition seemed to prevent high algal biomass at maximum PAR availability [51], [52]. We also observed structural differentiation (e.g., formation of filamentous streamers) of the biofilms as a response to fluctuating flow velocity [17], [53], but bulk biofilm biomass (including streamers) was still lower in the stochastic compared to the constant flow regime. After 42 days of growth, phototrophic biofilms achieved a consistently higher algal cell abundance in the constant than in the stochastic flow regime (Figure S3 in Supporting Information) – agreeing with the Chl-*a* values (Figure 3A). Algae community composition (ACC) was composed of 68 genera, clearly dominated by diatoms (mainly *Achnanthes* sp.). The resulting ordination (Figure 4B) pointed to clear shifts in ACC due to flow stochasticity and across the light gradient. Flow-driven and light-driven shifts were comparable in magnitude and occurred along separate ordination axes indicating independent compositional changes of ACC due to these two controls (see Text S1 in Supporting Information for more details).

**Figure 5:**
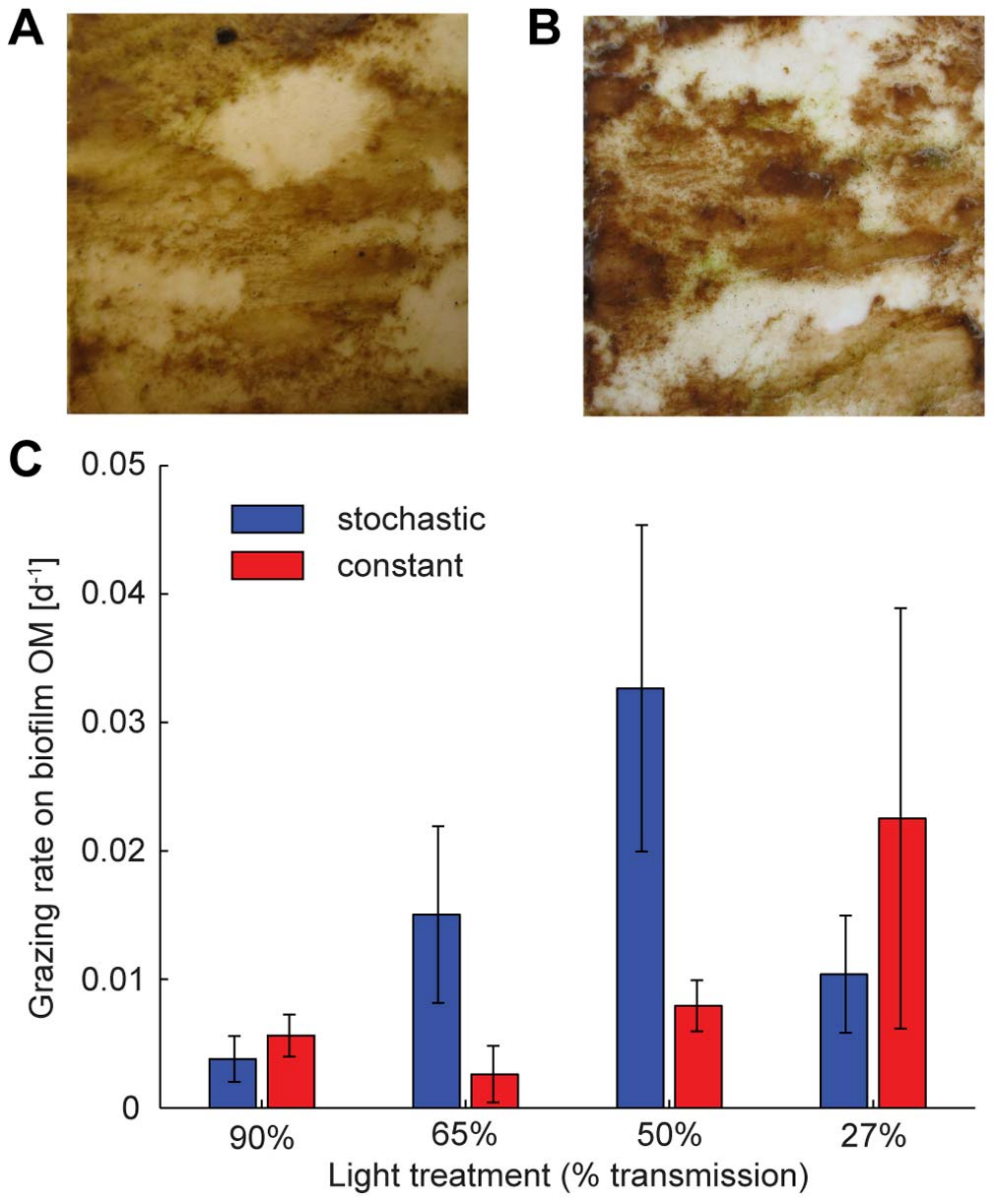
Invertebrate grazing dynamics. Grazing activity on sampled tiles, corresponding to (a) constant flow regime and 65% transmission of incident light on September 6^th^ and (b) stochastic flow regime and 27% transmission of incident light on September 10^th^, as representative examples of the observed grazing tracks. (c) *Ecdyonurus* grazing rate on biofilm organic matter [d^−1^] for each discharge and light treatment (mean ± SD). Two-way ANOVA on log-transformed values: discharge F_1,16_ = 5.13, P = 0.038; light F_3,16_ = 2.81, P = 0.073; discharge x light F_3,16_ = 1.03, P = 0.408. Blue and red bars refer to stochastic and constant discharge treatments, respectively.

### Invertebrate Grazing on Benthic Biofilms

After the initial phase without invertebrate grazers, larvae (n = 8) of the mayfly *Ecdyonurus* sp. were introduced to a flume segment yielding an areal abundance of 92 individuals m^−2^ and their grazing impact on biofilm biomass development over 17 days was quantified. Grazing rates by larval *Ecdyonurus* sp. were found to differ among the flow and light treatments (Figure 5C and Figure S4 in Supporting Information), with significantly higher values under the stochastic flow regime and at intermediate PAR availabilities (i.e., 65% and 50% transmission). To test whether resource quality, evaluated as the initial ACC, mediated the observed grazing rate patterns, we used a canonical correlative approach. This approach did not reveal any significant correlation between ACC and grazing rates (Figure S5 in Supporting Information). Also, flow- and light-associated canonical dimensions of ACC (i.e., changes of relative abundance patterns among 68 algae genera that are associated with the experimental treatments) were not correlated with grazing rate. The (non-significant) shifts of ACC potentially associated with grazing rate were correlated with the flow-driven shifts of ACC, but not with the equally strong light-driven shifts of ACC.

## Discussion

**Figure 6:**
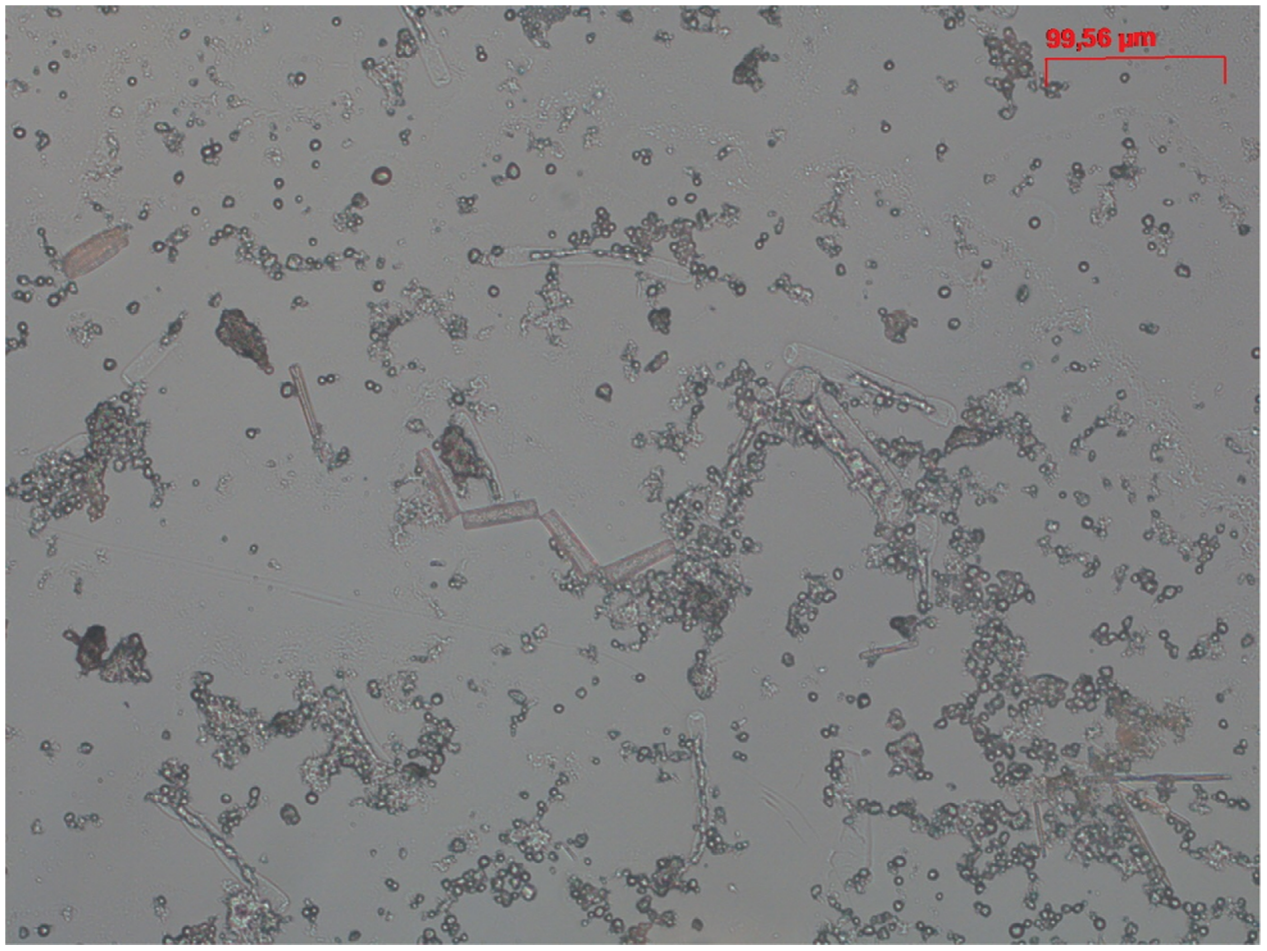
Microscopical analysis (Zeiss Axioimager) of the gut content of *Ecdyonurus* sp. larvae. The analysis did not allow to identify the composition of ingested algae (except Diatoma microcolonies). The content was largely digested already in the apical segment of the gut.

**Figure 7:**
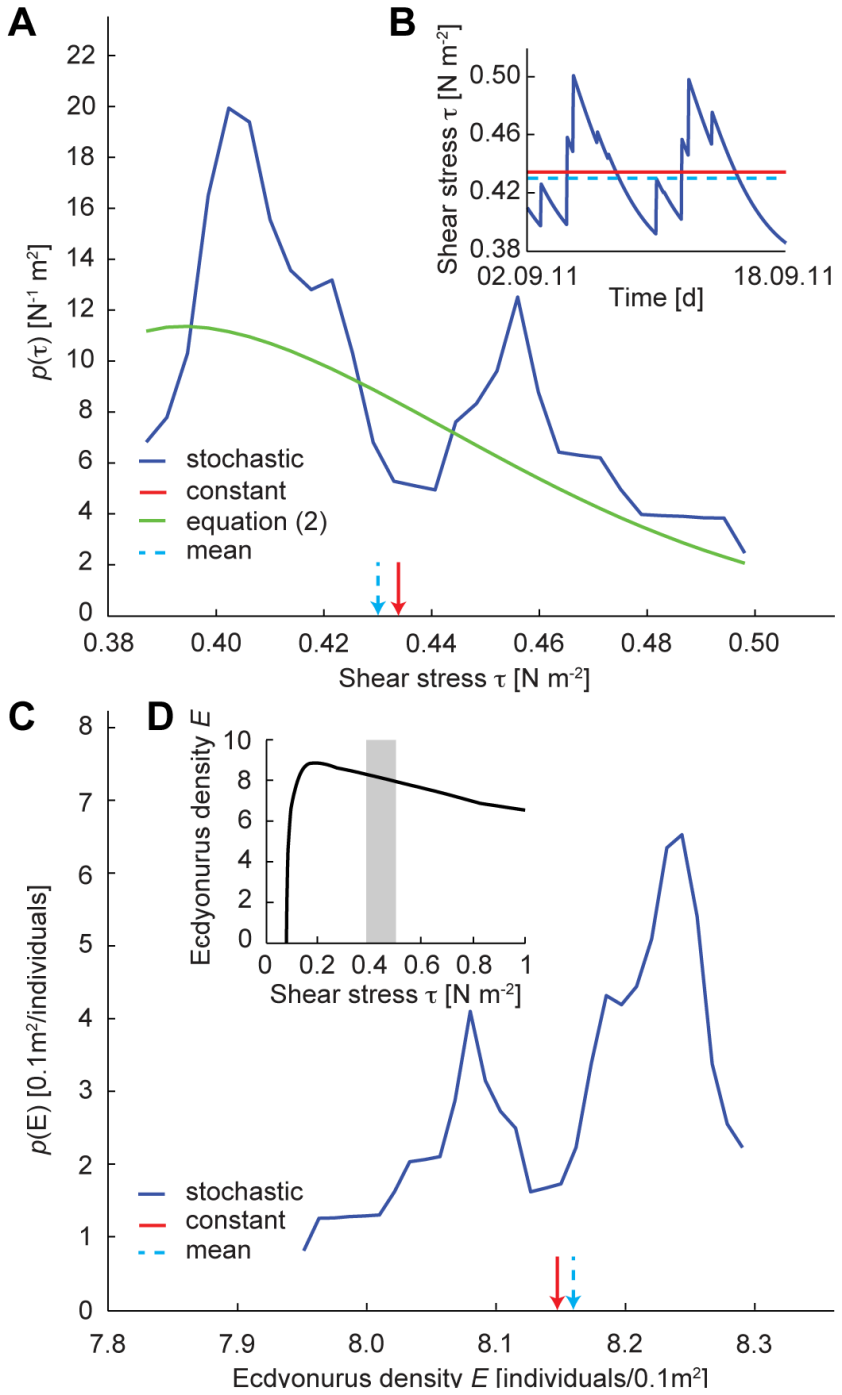
Suitability of experimental shear stress conditions for *Ecdyonurus* sp. larvae. (a) Probability distribution function (pdf) of shear stress, 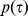: blue and green solid lines represent the empirical and the analytical pdfs, respectively; red solid arrow represents the shear stress 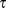 under constant discharge conditions; light blue dashed arrow represents the mean shear stress 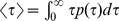 under stochastic discharge conditions. (b) Temporal sequence of shear stresses during grazing, from September 2^nd^ to September 18^th^ 2011: blue and red solid lines represent shear stresses in the stochastic and in the constant discharge treatment, respectively; light blue dashed line represents the mean shear stress 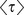 in the stochastic regime. (c) Probability distribution function of *Ecdyonurus* density 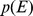 (from re-evaluation of existing field data [58]): blue solid line refers to the stochastic discharge treatment, red solid arrow refers to the constant discharge treatment, light blue dashed arrow represents the expected mean value under the stochastic regime. (d) *Ecdyonurus* habitat suitability curve (from re-evaluation of existing field data [58]): the grey area highlights the experimental range of shear stress.

The presented results, based on well controlled stream microcosms, help answering a longstanding question in ecology, namely how environmental variation mediates ecological processes and trophic interactions. Two possible factors may drive the contrasting patterns of invertebrate grazing on phototrophic biofilms between stochastic and constant flow regimes. First, both flow and light regimes may affect biofilm biomass and in particular their ACC and hence their palatability for grazers [16], [54]. However, the performed analysis strongly suggests that resource quantity and algal community composition did not affect the observed grazing pattern. Indeed, flow stochasticity can be considered as a common and strong control on both ACC and grazing, while it minimises the potential mediating role of ACC between flow stochasticity and grazing. This statistical finding was also supported by the obvious grazing tracks (characterised by almost complete clearance of biofilm) which the *Ecdyonurus* larvae left on the substratum (Figure 5A, B), suggesting a “bulldozer”-type of foraging [55], [56]. Gut analyses of selected larvae after the experiment did not allow the retrieval and identification of numerous algae, but revealed well-filled guts with largely unidentifiable masses. Given the autotroph nature of the cultivated biofilms with little detrital contents, this suggests high digestibility and generally high attractiveness of biofilms as food resource, which further corroborates the notion of non-selective feeding (Figure 6). Second, the near-bed hydraulic environment and, in particular, the bottom shear stress 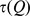, are well known to control both distribution and activity of benthic invertebrates in streams [21], [24]–[27], [57]. It is suggested here that stochastic flow, characterised by a wide distribution of bottom shear stresses, may offer more opportunities of reduced shear stress and therefore better foraging conditions for grazers than constant flow. From the temporal sequence in the stochastic flow regime (Figure 7B), the observed and analytical results for the shear stress probability distribution function, 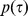, were compared (equation (2) and Figure 7A). Due to the positive skewness of the streamflow and shear stress probability distributions, the stochastic flow regime exhibited lower shear stresses than the constant flow environment during nearly 60% of the grazing phase of the experiment. Moreover, the average shear stress in the stochastic flow treatment was lower, thus resulting in an overall more favourable near-bed hydraulic environment for grazers.

To place these experimental findings in the context of natural populations, existing field data [58] on the relationship between bottom shear stress conditions and density of *Ecdyonurus* larvae in headwater streams were re-evaluated to inform about suitability of habitats in terms of shear stress. The *Ecdyonurus* larvae used in the experiment could be characterised by a hump-shaped habitat suitability curve (Figure 7D), which translates into an empirical probability distribution of suitability (Figure 7C). Indeed, this comparison suggests that the stochastic flow regime offers higher probabilities of more favourable hydraulic conditions. In other words, in the stochastic flow regime grazers could invest more energy into resource acquisition rather than into resistance to shear stress-induced erosion. The grazing rate pattern across flow and light treatments (Figure 5C) indicates interacting effects of flow (and related shear stress) regimes and PAR availability on biofilm biomass and grazing activity. *Ecdyonurus* larvae are known to have a strong diurnal rhythm and to graze preferentially during the night, a behaviour known to be directly controlled by light rather than by an internal clock [59]. Thus, in the constant flow regime, increasing grazing rates with decreasing PAR availability are supposedly a consequence of an increased grazing activity (i.e., foraging time) of *Ecdyonurus*. In the stochastic flow regime, increased foraging time would translate into increased chances to catch windows of opportunity with favourable shear stress conditions. This may lead to even higher grazing rates in terms of biofilm biomass removal and to an increasing flow regime effect on the grazing rate for the treatments from 90% to 50% transmission of incident light. At highest PAR availability (90% transmission), reduced foraging time may cloud flow regime effects, underscoring the interaction between flow regime and the control of light on grazing activity. At lowest PAR availability (27% transmission), resource limitation (low biofilm biomass) prevented increased grazing rates in the stochastic flow regime. Even if *Ecdyonurus* larvae could freely move across the flumes and find optimal windows of opportunity in terms of shear stress, they could not find sufficient biomass, as compared to intermediate light conditions. Thus, increased foraging time may not directly translate into increased grazing rates. Our results show that the maximum achievable grazing rate is a product of available biofilm biomass and grazer foraging activity, which are both interactively controlled by flow regime and PAR availability. At intermediate PAR availability (50% and 65% transmission), almost a 4-fold increase of the grazing rate is achieved in the stochastic compared to the constant streamflow treatment (Figure 5C). These findings thus suggest that temporal fluctuations of discharge and associated shear stress, together with PAR availability, modulate biofilm-grazer interactions. Compared to a constant flow environment, with identical mean discharge, a stochastic flow regime may offer grazers more opportunities to satisfy their resource needs, e.g., by allowing increased mobility across resource patches as these become depleted, and to avoid competitor encounter [21], [24]–[26].

Findings from our microcosm experiments suggest that elevated grazing rates under stochastic flow may even have consequences for ecosystem functioning and trophic transfer within food webs. In fact, based on OM removed by grazing, trophic transfer of organic carbon was estimated at 0.46 ± 0.39 mg C d^−1^ individual^−1^ and at 0.29 ± 0.19 mg C d^−1^ individual^−1^ in the stochastic and constant flow regime, respectively, under high PAR availability (90% transmission) (Table 2). Under low PAR availability (27% transmission), carbon fluxes averaged 0.79 ± 0.68 mg C d^−1^ individual^−1^ and 1.13 ± 1.27 mg C d^−1^ individual^−1^ in the stochastic and constant flow regime, respectively. Given the reported areal abundances of *Ecdyonurus* larvae in OSB and similar pre-alpine streams (Table S1 in Text S1 in Supporting Information), areal fluxes of organic carbon associated with biofilm grazing ranged from 0.13 g C m^−2^ d^−1^ to 0.54 g C m^−2^ d^−1^ under the stochastic flow regime and from 0.08 g C m^−2^ d^−1^ to 0.32 g C m^−2^ d^−1^ under the constant flow regime. This potential carbon flux induced by one model grazer is remarkable when compared to the gross primary production in headwater streams (0.72 ± 0.14 g C m^−2^ d^−1^; range: 0.02 to 5.62 g C m^−2^ d^−1^, n = 62) [60] and is potentially supported by accelerated turnover of biofilm-bound carbon. Higher turnover, i.e., increased net productivity by grazed biofilms, may be induced by removal of senescent biofilm biomass, enhanced diffusion rates and increased light availability, as well as nutrient subsidies [15], [48]–[50]. We note that our OC flux estimates are not only due to consumption by *Ecdyonurus*, but also include OC dislodged from the biofilm by bioturbation [15], [47]. The resulting OC flux enters the water column in particulate form and is exported to downstream food webs, where it constitutes an important food resource for collectors and gatherers, the most abundant FFG in streams like the OSB and larger rivers.

Our findings thus unravel small-scale trophic processes and how these may change as a streamflow regime is altered. Possible shortcomings (e.g., absence of predation) of our conceptual model may be recognised when findings derived from this model grazer system are transferred to real ecosystems [3]. However, the microcosm experiments with model organisms employed here, because of their rigorous control and reproducibility, are well suited to clarify mechanisms otherwise not accessible by field observations. Such microcosms are often even used to address ecological problems at a global scale [61].

The increasing intensity of water resource management, associated with securing water supplies, agricultural irrigation, hydropower production and flood protection, implies various alterations to natural streamflow regimes, which, in turn, may have severe effects on fluvial ecosystem structure and function [62]–[64]. Indeed future environmental impact criteria, effective management and restoration of fluvial ecosystems should include assessments of impacts on ecosystem processes. Our experiment provides a first and important evidence of hitherto undisclosed effects of flow regime changes on ecosystem functioning, and suggests that alterations simply maintaining a minimum constant flowrate as an environmentally conscious management strategy is inadequate to fully preserve ecosystem integrity.

## Supporting Information

Figure S1
**Biofilm Chl-** ***a*** **[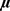
g cm^−2^] temporal dynamics (mean ± SD).** Left panels refer to the stochastic discharge treatment. (a), (b), (c), (d), refer to 90%, 65%, 50% and 27% transmission of incident light, respectively. Dark blue triangles and solid lines, and light blue circles and dashed lines represent biomass under ungrazed and grazed conditions, respectively. Analogously, right panels refer to the constant discharge treatment. Red triangles and solid lines, and orange circles and dashed lines represent biomass under ungrazed and grazed conditions, respectively. Black arrows indicate grazers’ inclusion in the flumes (on September 2^nd^).(TIF)

Figure S2 **Biofilm OM [mg cm^−2^] temporal dynamics (mean ± SD).** Left panels refer to the tochastic discharge treatment. (a), (b), (c), (d), refer to 90%, 65%, 50% and 27% transmission of incident light, respectively. Dark blue triangles and solid lines, and light blue circles and dashed lines represent biomass under ungrazed and grazed conditions, respectively. Analogously, right panels refer to the constant discharge treatment. Red triangles and solid lines, and orange circles and dashed lines represent biomass under ungrazed and grazed conditions, respectively. Black arrows indicate grazers’ inclusion in the flumes (on September 2^nd^).
(TIF)

Figure S3 **Biofilm algal cell abundance and community composition for each discharge and light treatment.**
(TIF)

Figure S4 ***Ecdyonurus*** **grazing rate on biofilm Chl-** ***a*** **[d^−1^] for each discharge and light treatment (mean ± SD).** Two-way ANOVA on log-transformed data: discharge F_1,16_ = 9.64, P = 0.007; light F_3,16_ = 3.92, P = 0.028; discharge×light F_3,16_ = 2.31, P = 0.116. Blue and red bars refer to stochastic and constant discharge treatments, respectively.
(TIF)

Figure S5 **Correlations among light-driven, flow-driven and potentially grazing-associated shifts of autotrophic community composition of benthic biofilms.** Each axis represents one canonical dimension identified by canonical analysis of principal coordinates run on the Bray-Curtis dissimilarity matrix with flow and light or grazing rate as constraint(s).
(TIF)

Figure S6 **Plan of the experimental setup.** For this experiment we used 24 out of 36 flumes (12 for each discharge treatment).
(TIF)

Figure S7 **Sections of the experimental setup.** (a) Section A–A; (b) Section B–B.
(TIF)

Figure S8 **Header tank: particular.** (a) Portion from Section A–A; (b) Section C–C; (c) Section D–D; (d) Section E–E; (e) Section F–F.
(TIF)

Figure S9 **Small tank and flumes: particular.** (a) Portion from Section A–A; (b) Section G–G; (c) Section H–H; (d) Section L–L.
(TIF)

Figure S10 **Flume light sequence.**
(TIF)

Figure S11 **Biofilm growth rate estimation.** Blue circles and red triangles represent measured biomass values from stochastic and constant discharge treatments, respectively. Light blue circle and orange triangle represent the average value of triplicate biomass measurements on September 1^st^ for stochastic and constant discharge treatments, respectively. The slope of the line corresponds to the growth rate. The plot refers to the light condition characterised by 65% transmission of incident light.
(TIF)

Figure S12 **Grazing rate estimation.** Blue triangles and light blue circles represent measured biomass values from the stochastic discharge treatments under ungrazed and grazed conditions, respectively. The slope of the blue line represents the growth rate, while the slope of the light blue line represents the difference between the growth rate and the grazing rate. The plot refers to the light condition characterised by 65% transmission of incident light.
(TIF)

Text S1 (PDF)
