# Supplementary figures and images for: Hydrologic Variability Affects Invertebrate Grazing on Phototrophic Biofilms in Stream Microcosms
Source: PLoS One. 2013 Apr 16;8(4):e60629. doi: 10.1371/journal.pone.0060629 (PMC3628795; doi:10.1371/journal.pone.0060629)

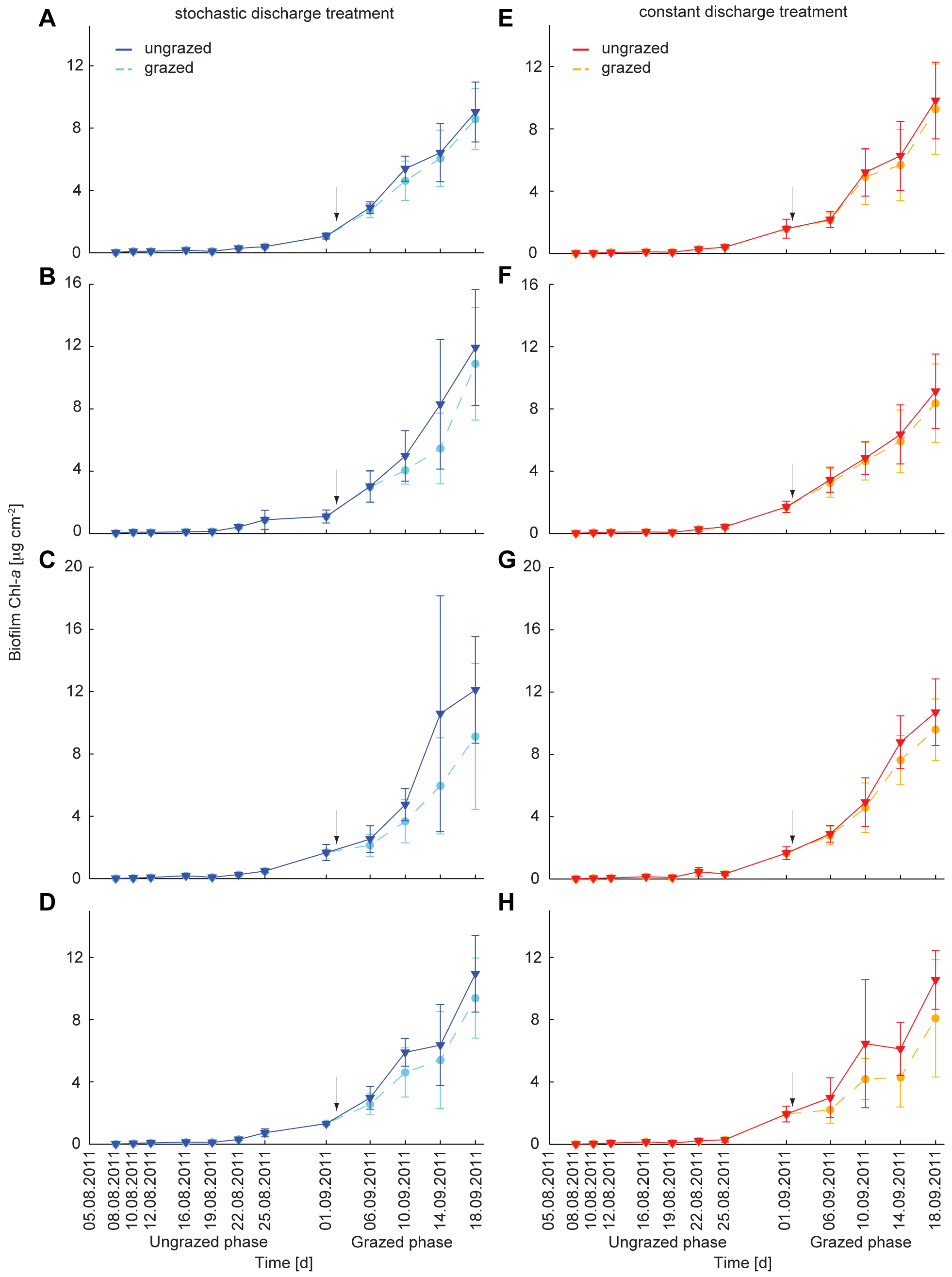

Supplement: Figure S1 — Biofilm Chl- a [g cm−2] temporal dynamics (mean ± SD). Left panels refer to the stochastic discharge treatment. (a), (b), (c), (d), refer to 90%, 65%, 50% and 27% transmission of incident light, respectively. Dark blue triangles and solid lines, and light blue circles and dashed lines represent biomass under ungrazed and grazed conditions, respectively. Analogously, right panels refer to the constant discharge treatment. Red triangles and solid lines, and orange circles and dashed lines represent biomass under ungrazed and grazed conditions, respectively. Black arrows indicate grazers’ inclusion in the flumes (on September 2nd). (TIF) [file pone.0060629.s001.tif]

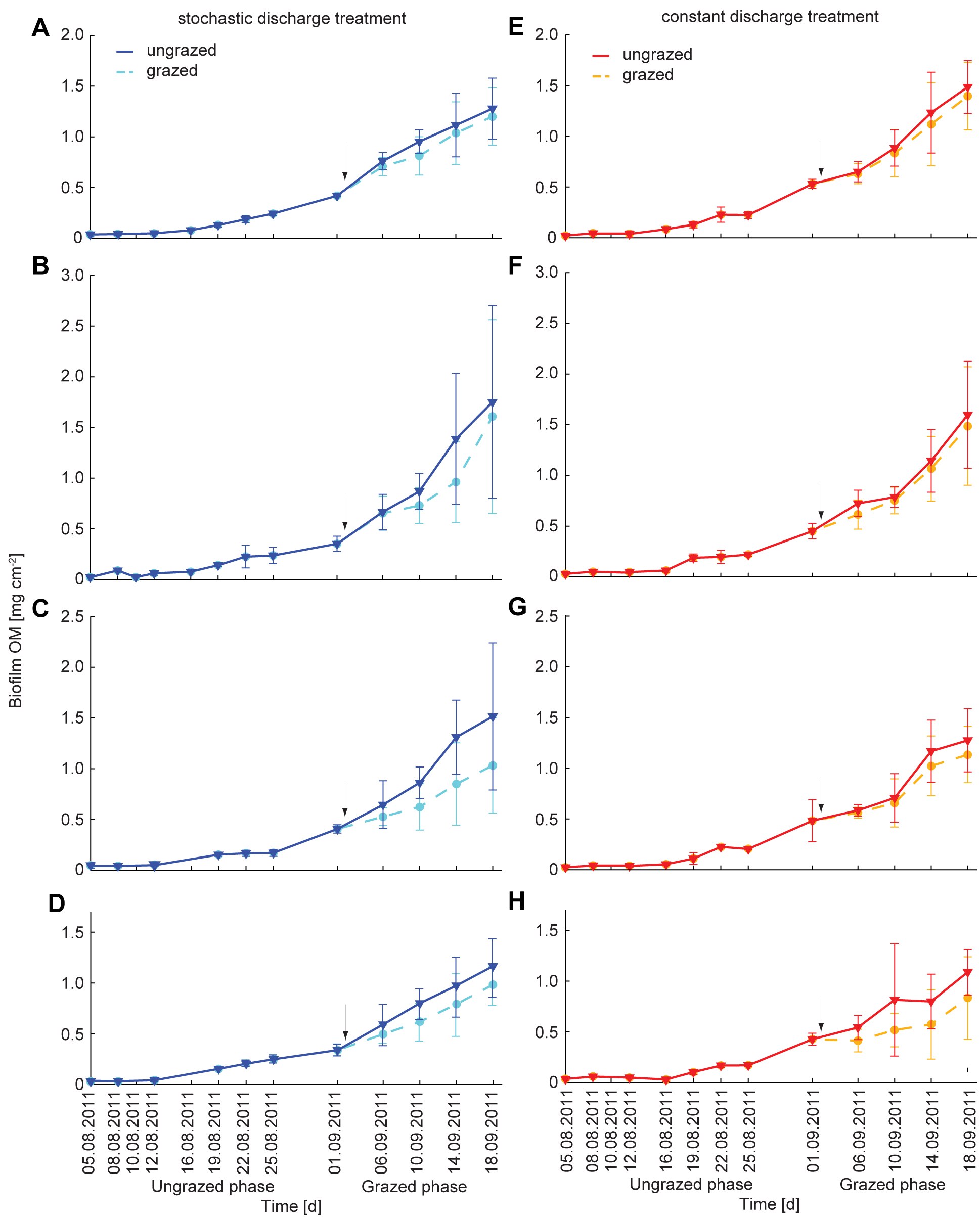

Supplement: Figure S2 — Biofilm OM [mg cm−2] temporal dynamics (mean ± SD). Left panels refer to the tochastic discharge treatment. (a), (b), (c), (d), refer to 90%, 65%, 50% and 27% transmission of incident light, respectively. Dark blue triangles and solid lines, and light blue circles and dashed lines represent biomass under ungrazed and grazed conditions, respectively. Analogously, right panels refer to the constant discharge treatment. Red triangles and solid lines, and orange circles and dashed lines represent biomass under ungrazed and grazed conditions, respectively. Black arrows indicate grazers’ inclusion in the flumes (on September 2nd). (TIF) [file pone.0060629.s002.tif]

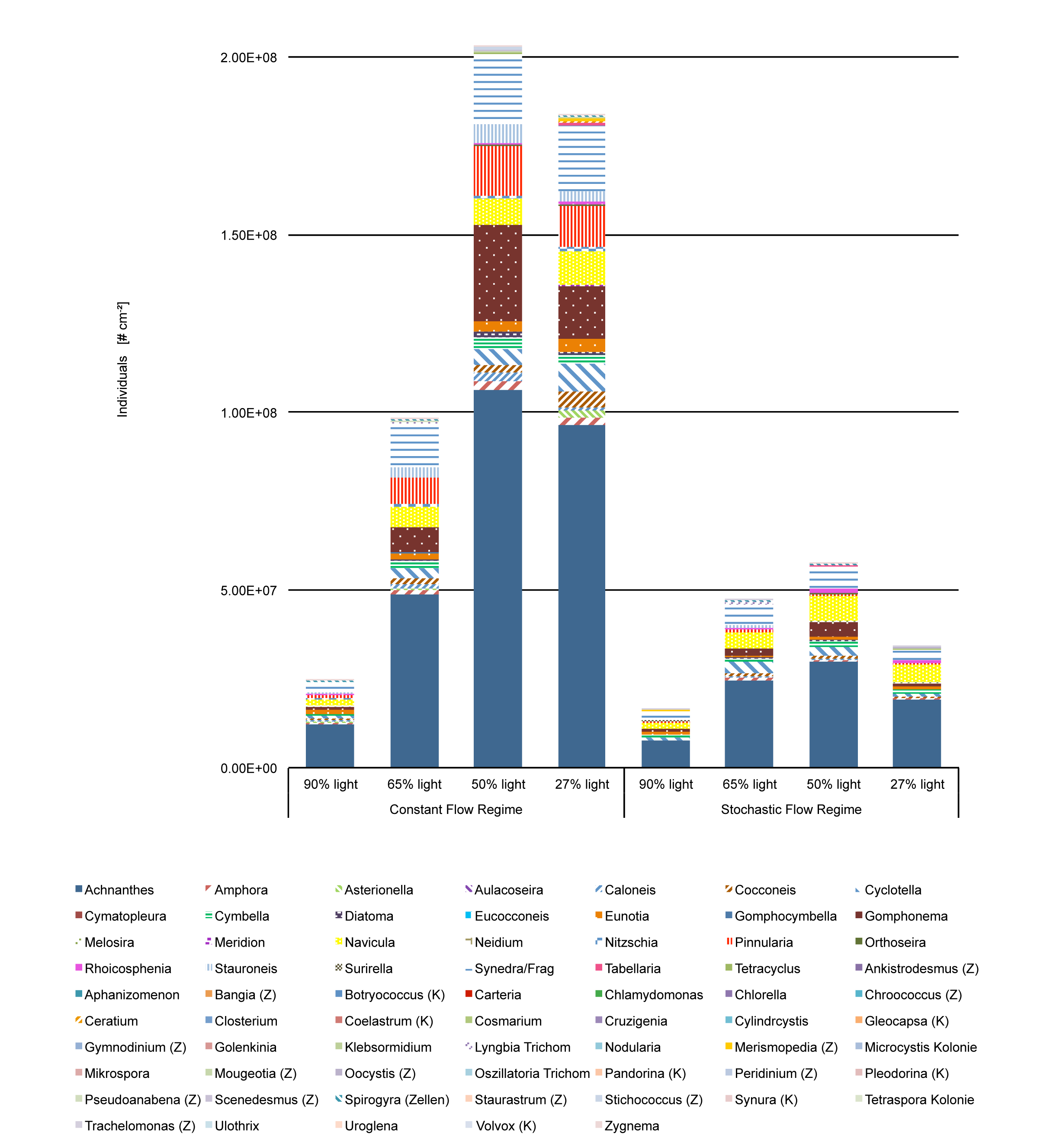

Supplement: Figure S3 — Biofilm algal cell abundance and community composition for each discharge and light treatment. (TIF) [file pone.0060629.s003.tif]

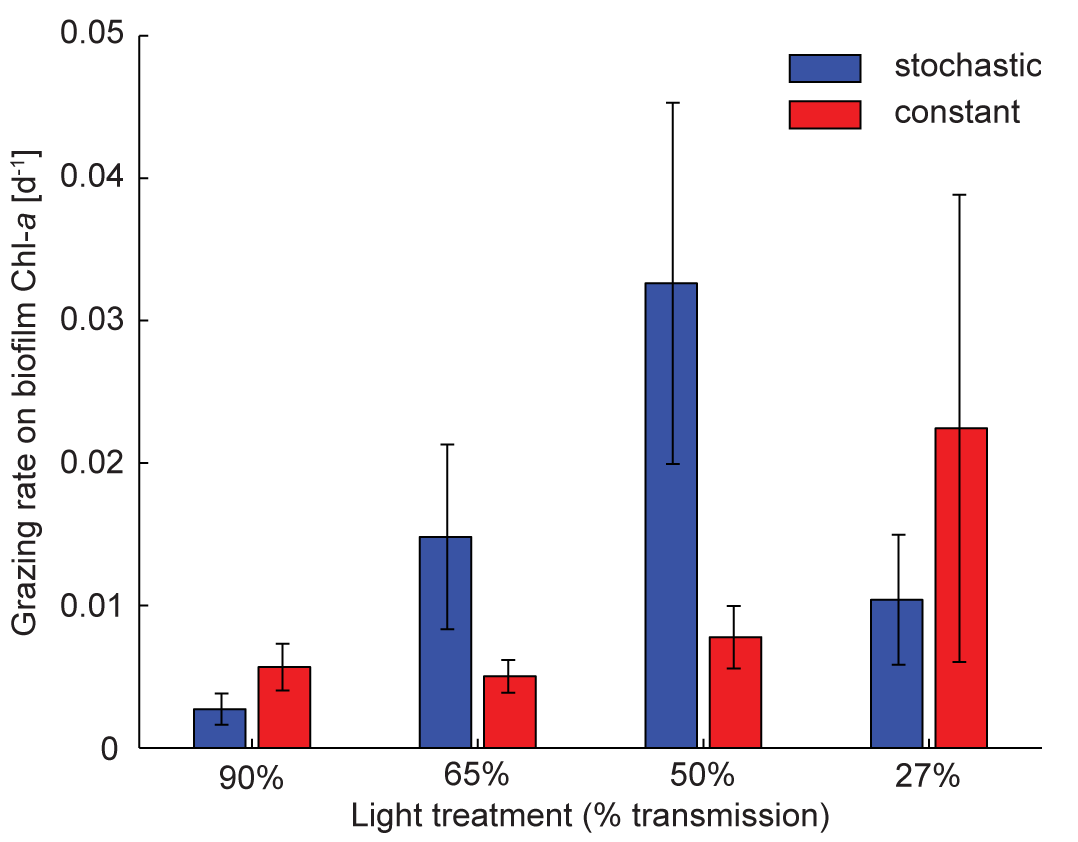

Supplement: Figure S4 — Ecdyonurus grazing rate on biofilm Chl- a [d−1] for each discharge and light treatment (mean ± SD). Two-way ANOVA on log-transformed data: discharge F1,16 = 9.64, P = 0.007; light F3,16 = 3.92, P = 0.028; discharge×light F3,16 = 2.31, P = 0.116. Blue and red bars refer to stochastic and constant discharge treatments, respectively. (TIF) [file pone.0060629.s004.tif]

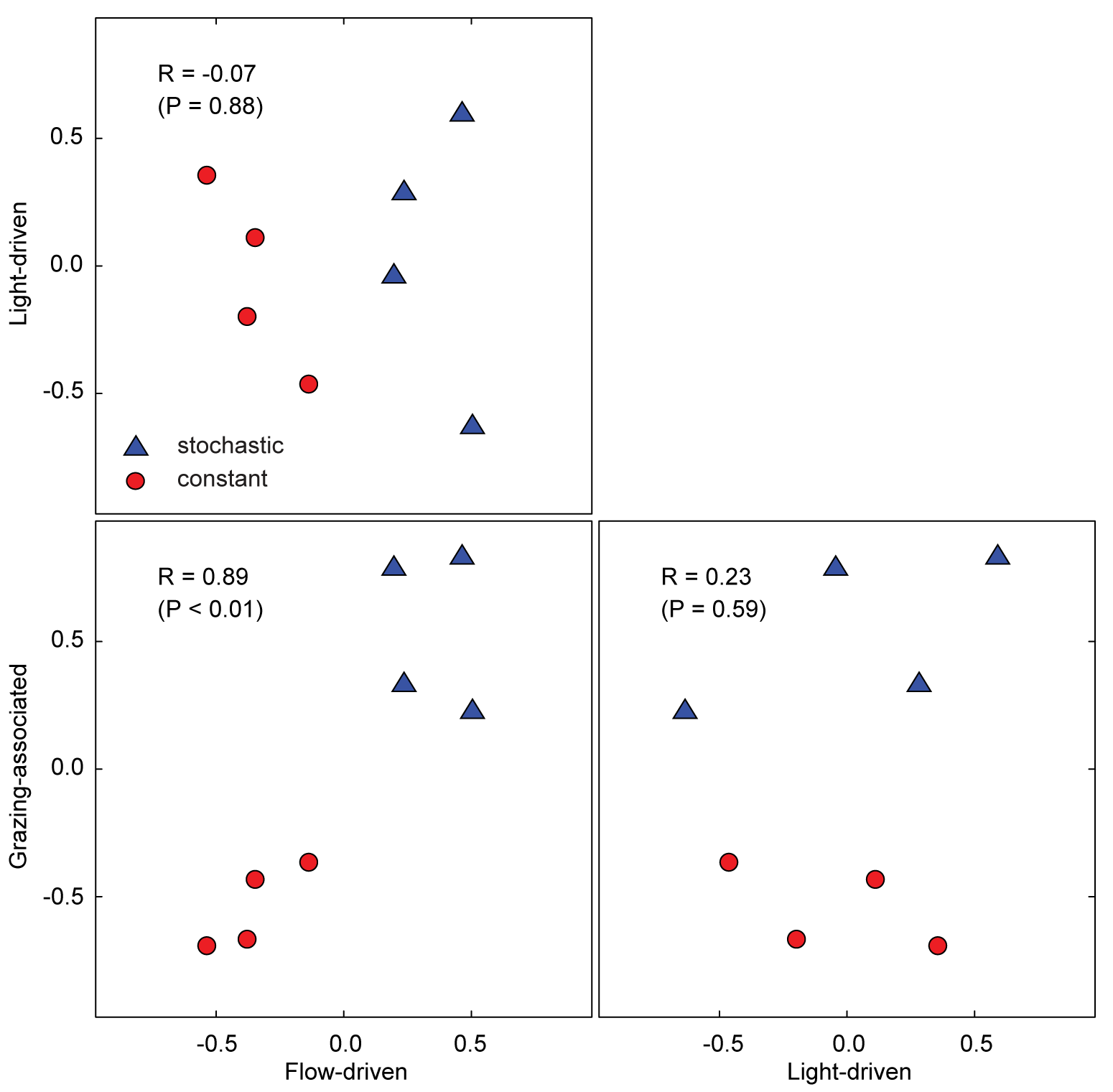

Supplement: Figure S5 — Correlations among light-driven, flow-driven and potentially grazing-associated shifts of autotrophic community composition of benthic biofilms. Each axis represents one canonical dimension identified by canonical analysis of principal coordinates run on the Bray-Curtis dissimilarity matrix with flow and light or grazing rate as constraint(s). (TIF) [file pone.0060629.s005.tif]

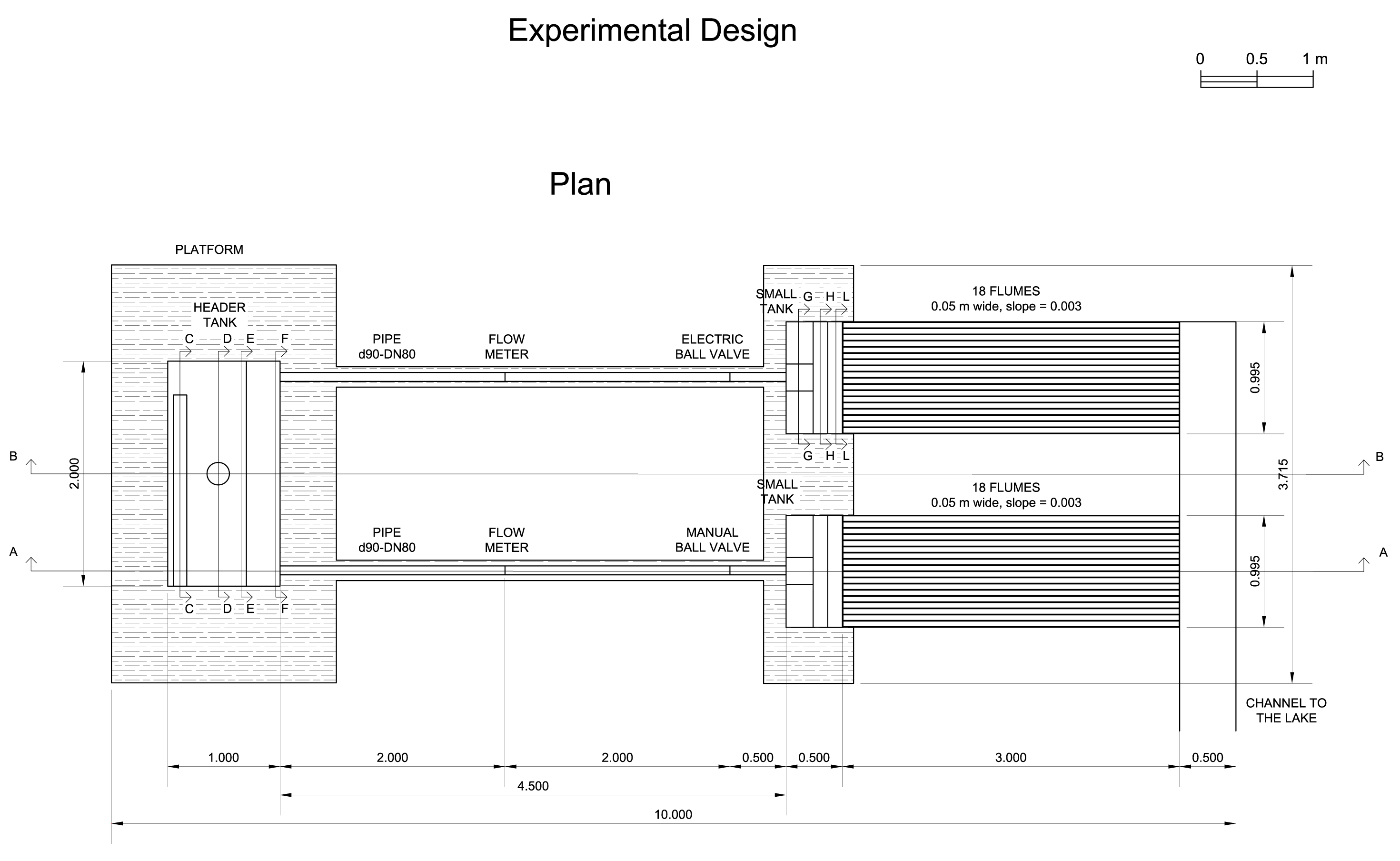

Supplement: Figure S6 — Plan of the experimental setup. For this experiment we used 24 out of 36 flumes (12 for each discharge treatment). (TIF) [file pone.0060629.s006.tif]

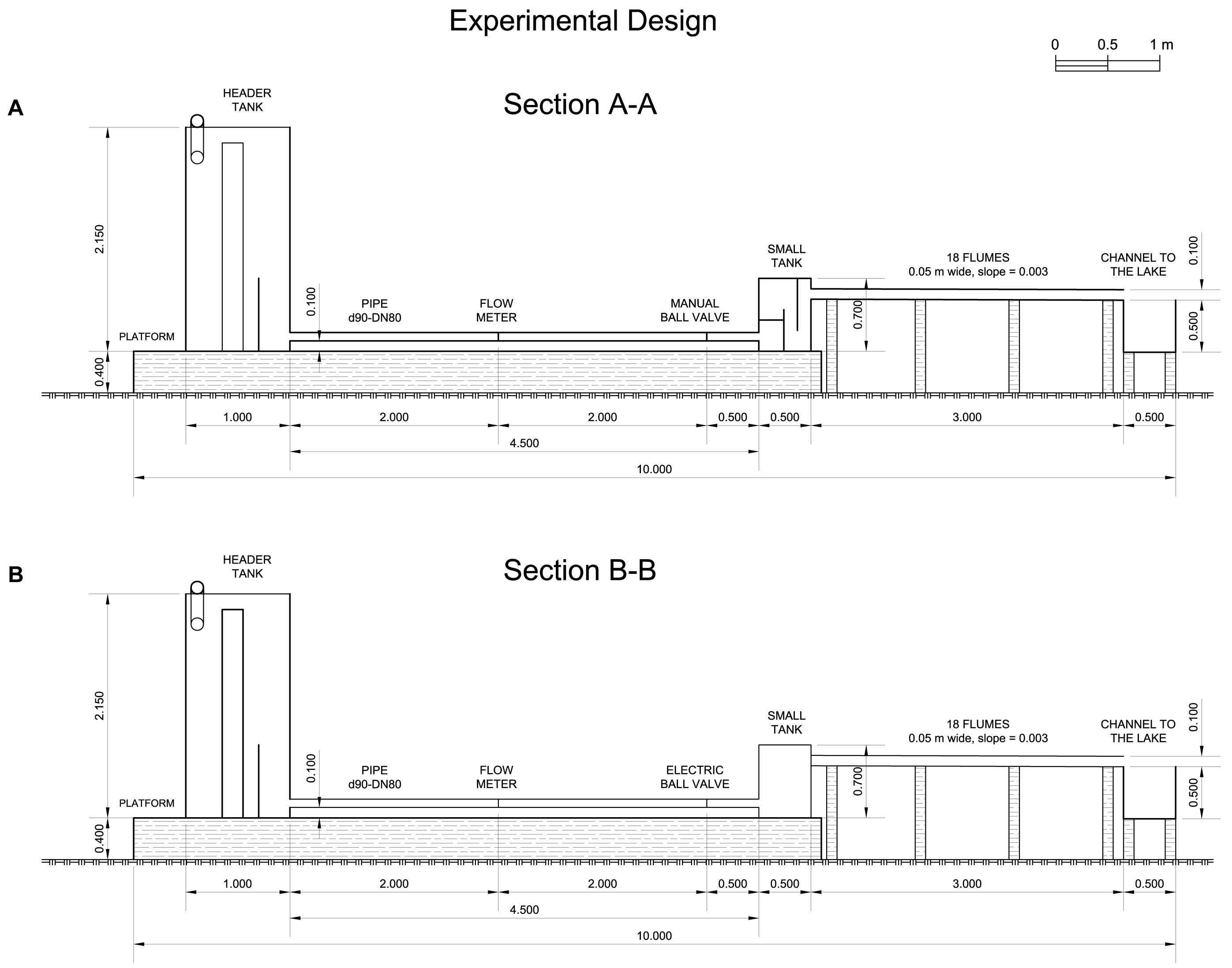

Supplement: Figure S7 — Sections of the experimental setup. (a) Section A–A; (b) Section B–B. (TIF) [file pone.0060629.s007.tif]

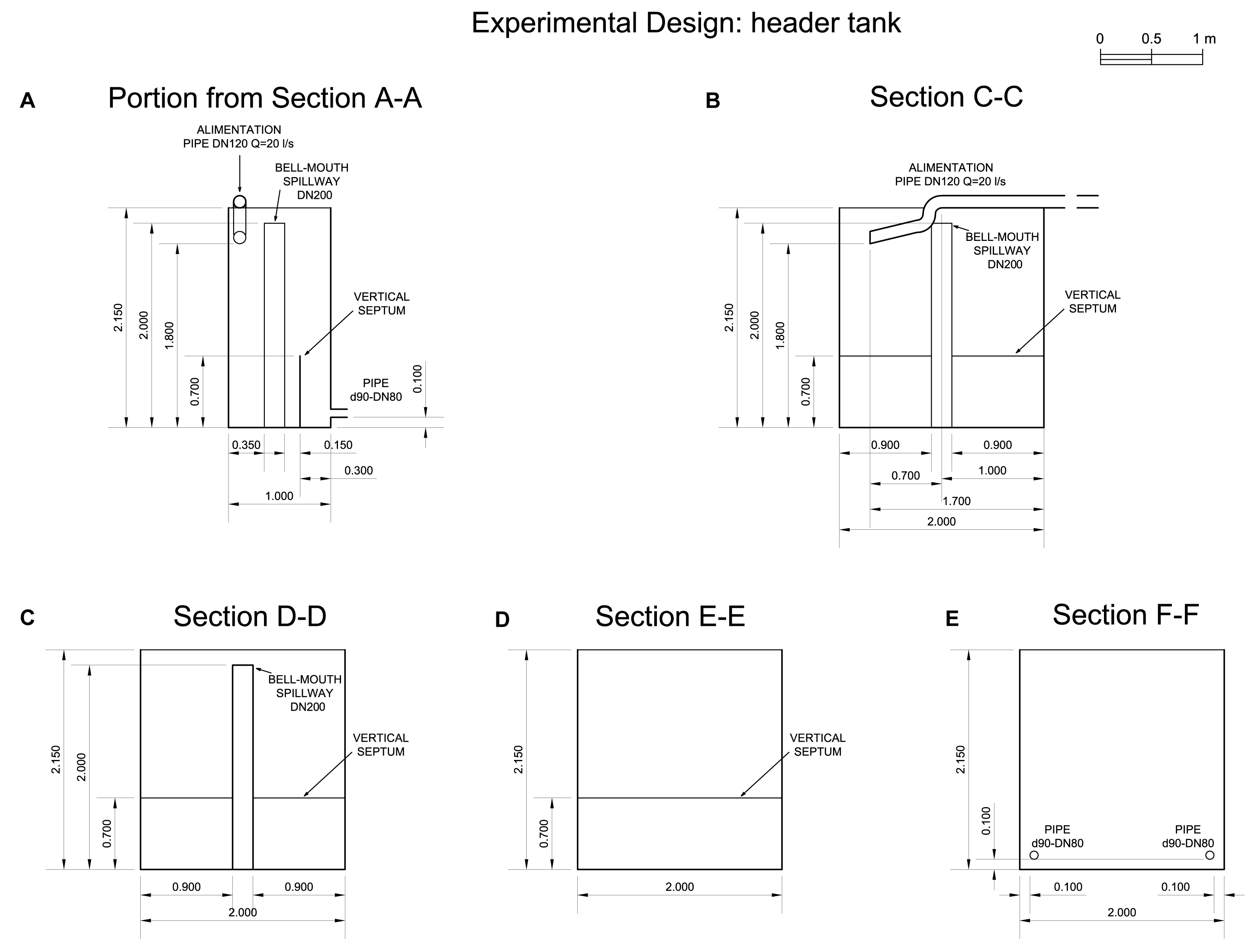

Supplement: Figure S8 — Header tank: particular. (a) Portion from Section A–A; (b) Section C–C; (c) Section D–D; (d) Section E–E; (e) Section F–F. (TIF) [file pone.0060629.s008.tif]

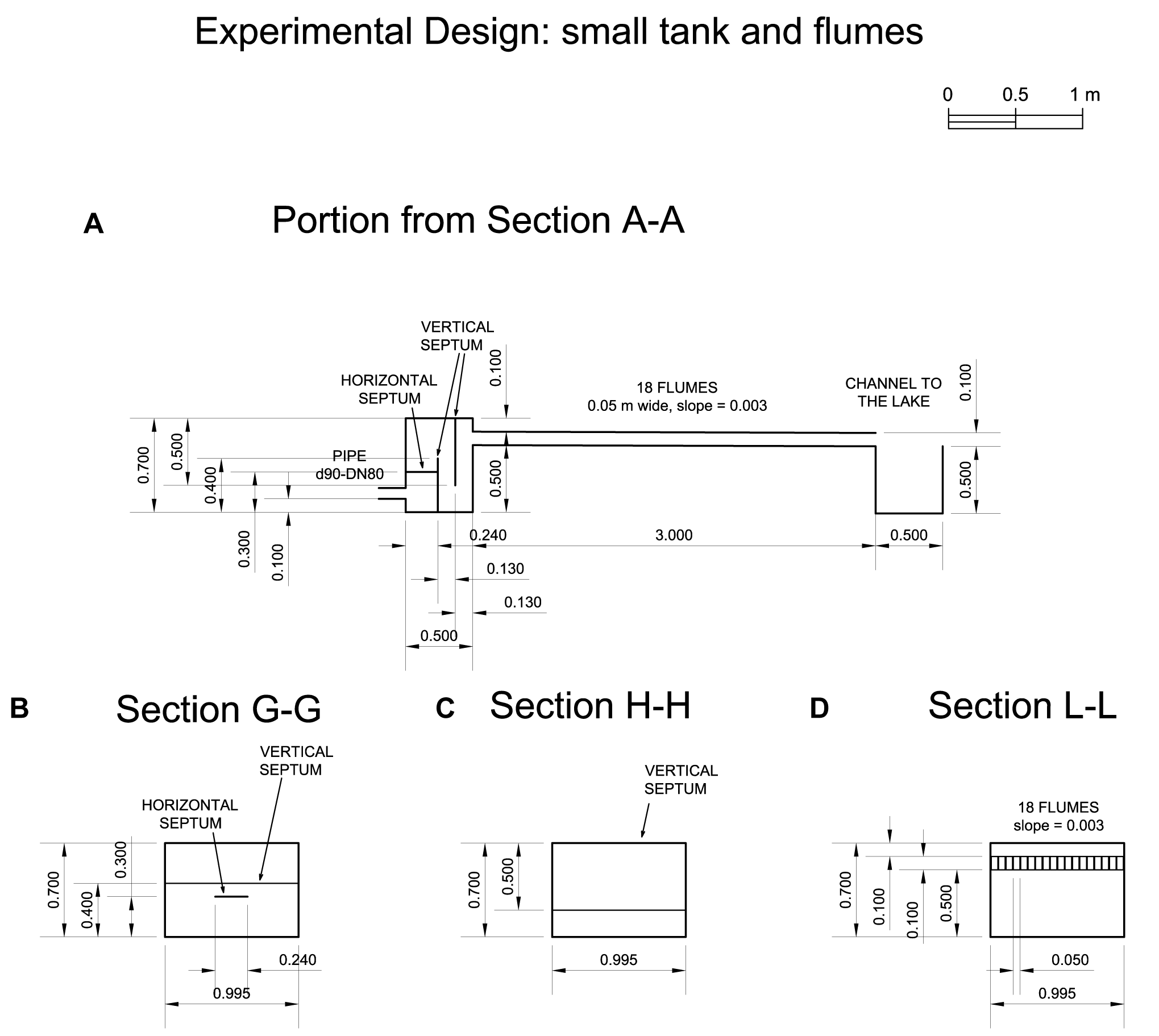

Supplement: Figure S9 — Small tank and flumes: particular. (a) Portion from Section A–A; (b) Section G–G; (c) Section H–H; (d) Section L–L. (TIF) [file pone.0060629.s009.tif]

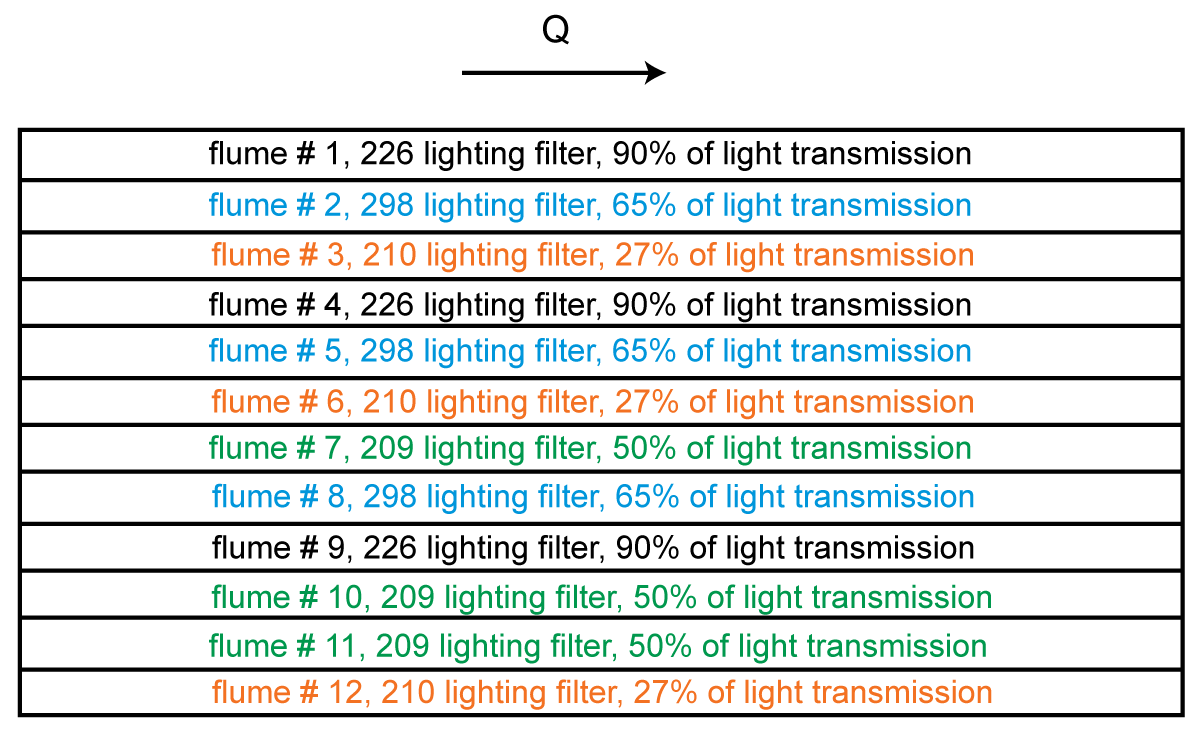

Supplement: Figure S10 — Flume light sequence. (TIF) [file pone.0060629.s010.tif]

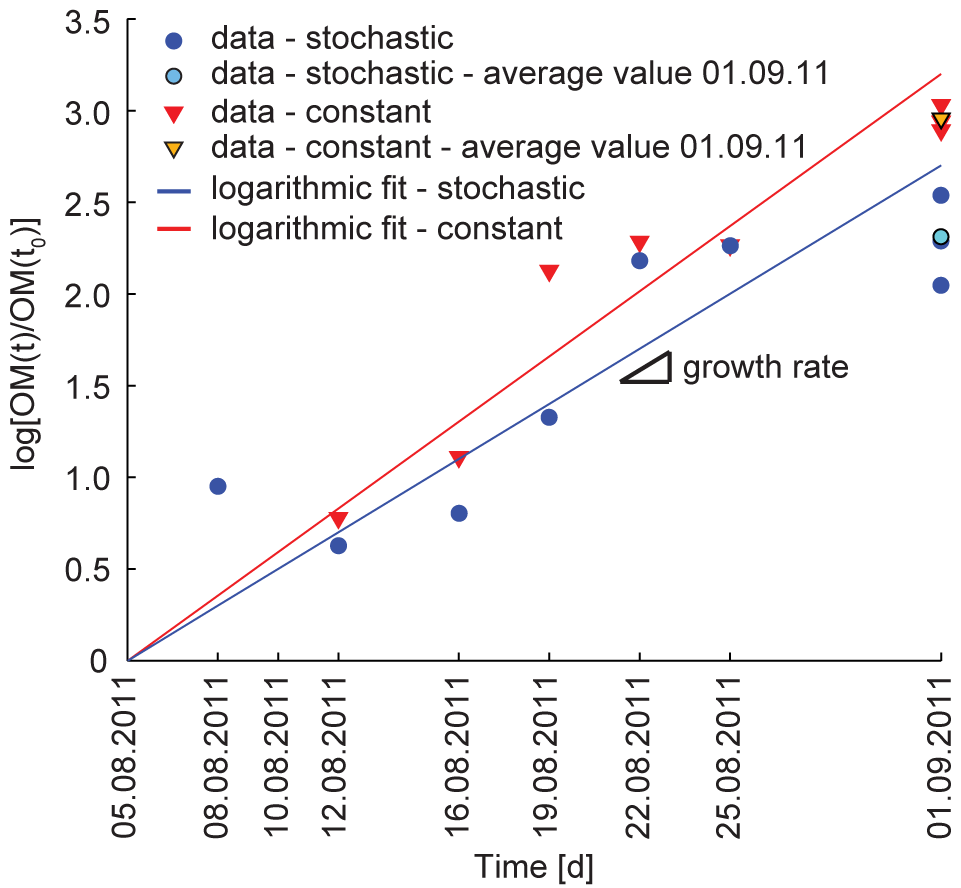

Supplement: Figure S11 — Biofilm growth rate estimation. Blue circles and red triangles represent measured biomass values from stochastic and constant discharge treatments, respectively. Light blue circle and orange triangle represent the average value of triplicate biomass measurements on September 1st for stochastic and constant discharge treatments, respectively. The slope of the line corresponds to the growth rate. The plot refers to the light condition characterised by 65% transmission of incident light. (TIF) [file pone.0060629.s011.tif]

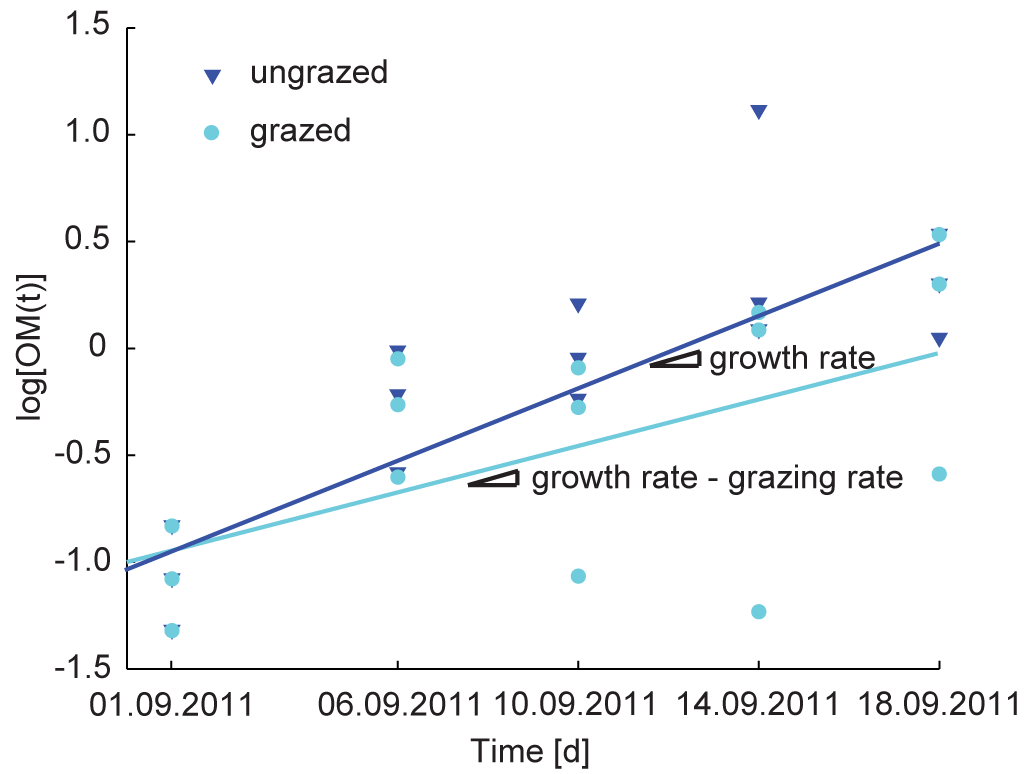

Supplement: Figure S12 — Grazing rate estimation. Blue triangles and light blue circles represent measured biomass values from the stochastic discharge treatments under ungrazed and grazed conditions, respectively. The slope of the blue line represents the growth rate, while the slope of the light blue line represents the difference between the growth rate and the grazing rate. The plot refers to the light condition characterised by 65% transmission of incident light. (TIF) [file pone.0060629.s012.tif]
