# Supplementary material for: Hydrologic Variability Affects Invertebrate Grazing on Phototrophic Biofilms in Stream Microcosms
Source: PLoS One. 2013 Apr 16;8(4):e60629. doi: 10.1371/journal.pone.0060629 (PMC3628795; doi:10.1371/journal.pone.0060629)
Supplement: Text S1 — (PDF) [file pone.0060629.s013.pdf]

Supporting Information for:
Hydrologic variability affects invertebrate grazing on phototrophic biofilms in stream microcosms

Serena Ceola[1,2], Iris Hödl[3], Martina Adlboller[3], Gabriel Singer[3], Enrico Bertuzzo[1], Lorenzo Mari[1,4], Gianluca Botter[5], Johann Waringer[3], Tom J. Battin[3,6], Andrea Rinaldo[1,5,*]

1 Laboratory of Ecohydrology (ECHO/IEE/ENAC), École Polytechnique Fédérale de Lausanne, 1015 Lausanne, Switzerland

2 Dipartimento di Ingegneria Civile, Chimica, Ambientale e dei Materiali, Università di Bologna, 40136 Bologna, Italy

3 Department of Limnology, University of Vienna, 1090 Vienna, Austria

4 Dipartimento di Elettronica, Informazione e Bioingegneria, Politecnico di Milano, 20133 Milano, Italy

5 Dipartimento di Ingegneria Civile, Edile ed Ambientale, Università degli Studi di Padova, 35131 Padova, Italy

6 WasserCluster Lunz, Interuniversity Center for Aquatic Ecosystem Research, 3293 Lunz am See, Austria

$*$ E-mail: andrea.rinaldo@epfl.ch

Supplementary material and methods

Experimental setup

The experiment was conducted at the WasserCluster Lunz, in Lunz am See (Austria, 47.86° N, 15.05° E), between July and September 2011. The design consisted of two Plexiglas slabs (one for each discharge treatment), both 3 m long, 0.995 m wide, 0.1 m deep, with a slope of 0.003, and internal partitions to create 18 flumes 0.05 m wide, that were operated in once-through flow mode (Figure S6 in Supporting Information). Half (i.e., 18) of the flumes experienced a stochastic discharge condition, and the remaining half a constant one (Figure 1A). Only 24 out of 36 flumes were used for this experiment. 62 low-porosity, unglazed ceramic tiles (47.6 ± 0.2 mm long and wide, 9.0 ± 0.1 mm thick) paved each flume and constituted a suitable substratum for biofilm growth and grazing activity of mayfly larvae [1]. Water was supplied through a submerged pump, with temperature values between 10.5°C and 13.9°C, an optimal range for the adopted grazing species. The pumped water flowed into a header tank, from where it moved into two pipes (for stochastic and constant discharge treatments) placed at the bottom of the tank, and then entered two smaller tanks that supplied the flumes (Figure S6 and S7 in Supporting Information). The designed setup ensured that all flumes belonging to the same discharge treatment experienced identical hydraulic conditions, as the flume water level equalled the water level of the small tank. The header tank (4.3 m^3, 2 m long, 1 m wide, 2.15 m deep) received the pumped discharge of 20 $l\ s^{-1}$ through a slightly tilting pipe (Diameter Nominal 120 mm, DN120), whose outlet was placed at a depth of 1.80 m from the bottom of the tank (Figure S8A, B in Supporting Information). Inside the header tank (i) a morning glory spillway (DN200), whose overflow was placed at a depth of 2 m above the tank bottom, removed the exceeding water (Figure S8A, B, C in Supporting Information), and (ii) a vertical septum (0.7 m deep from the bottom) slowed down water velocity approaching the pipes, whose lower edge was placed 10 cm above the tank bottom (Figure S8A, D, E in Supporting Information). Two PVC-U pipes (external diameter 90 mm - Diameter Nominal 80 mm, d90-DN80), one for the stochastic discharge treatment and the other for the constant discharge treatment, were used. A propeller flow meter (+GF+ Signet 2536 Rotor-X Paddlewheel Flow Sensor) was placed in each pipe at a distance of 2 m from the header tank to record the flowing discharge (Figure S6, S7 in Supporting Information). A ball valve (d63-DN50) with suitable joints (d90-75 and d75-63) was placed nearly 2 m (i.e., 40 diameters/widths) downstream of the flow meter (Figure S6 in Supporting Information). In

particular, a computer-controlled electric ball valve (+GF+ type 130 - 100-230 V, with electric actuator EA21 and position signalisation 4-20 mA combined with the positioner PE25) was used to realise a controlled stochastic discharge sequence, while a manual ball valve (+GF+ type 546 PVC-U) was used to regulate the constant discharge. Two small tanks (0.35 m^3, each 0.995 m long, 0.5 m wide, 0.7 m deep) supplied the Plexiglas flumes glued on them (Figure S6, S7, S9 in Supporting Information). Inside the small tanks, a system of horizontal and vertical septa (Figure S9A, B, C in Supporting Information) reduced incoming water velocity, which was very high due to the high pressure conditions in the pipes. The various components of the setup were covered to avoid wind-blown inputs (e.g., rain, leaves, insects). At the flume inlet, a net was placed to regulate flow, to enhance uniform flow conditions, and to prevent grazers from crawling against the current and into the tank. To sustain water levels and to confine grazers in the upstream portion of the flumes, two additional nets were located 1.75 m downstream and at the flume outlet. The flume outlet was open and water freely flowed into a small channel.

Experimental procedure

Daily analysis consisted of measurements of water level in each flume and water temperature both in the flumes and in the header tank. Discharge was measured continuously by the flow meters. During the initial experimental phase in which grazers were excluded (from July 22nd to September 1st), one tile from each flume was sampled in intervals of two to seven days. The tiles were selected in the final part of the flume, moving upstream and avoiding the last 25 cm, in which uniform hydraulic conditions were not well established. A fresh, uncolonised tile was replaced at the sampled position. During the grazed phase (from September 2nd to September 18th), three tiles from the upstream part of each flume were sampled in intervals of four days. For each sampling day one tile from the upper, intermediate and lower part of the upstream flume segment were removed, avoiding the first 35 cm and last 25 cm, possibly characterised by non uniform flow conditions. The sampled tiles were replaced by unsampled but colonised tiles from the downstream sector, and the latter were replaced by fresh, uncolonised tiles.

Each sampled tile was photographed before complete removal of biofilm biomass with a sterile razor blade. A suspension of scraped biofilm biomass and MilliQ water was prepared in a screw-cap tube and then processed with a vortex for 10 s to homogenise it. A sonication at amplitude of 10% from one to two minutes was carried out to disrupt cell aggregates. Subsamples of the well-mixed suspension were filtered through glass microfiber binder free filters Whatman^{TM} GFC (1.2 μm) to determine ash-free dry mass (AFDM) and Chlorophyll-a (Chl-a) concentrations. AFDM is a proxy for the total organic matter (OM) of biofilm (i.e., microbes, algae, diatoms, extracellular polysaccharide), while Chl-a is a proxy for algae biomass. The OM filter was put in the drying oven at 70°C for 24 hours, moved to the desiccator for 24 hours to preserve its mass and then weighed for dry mass. The filter was subsequently put in the muffle furnace at 450°C for 4 hours, again in the desiccator, and finally weighed for ash mass. OM [mg cm^{-2}] has been determined as [2] $\mathrm{OM}=(\mathrm{W_a}\text{-}\mathrm{W_{ash}})\mathrm{ratio_{OM}}/A_{\mathrm{tile}}$ where $\mathrm{W_a}$ [mg] is the sum of filter weight and dried biofilm on filter, $\mathrm{W_{ash}}$ [mg] is the sum of filter weight and material on filter after ashing, $\mathrm{ratio_{OM}}$ is the ratio between the total suspension volume (from whole scraped biofilm biomass) and the suspension volume used for the OM filter, and $\mathrm{A_{tile}}$ [cm^2] is the area of the tile. The Chl-a filter was folded and closed in an aluminium foil, and then put in a -18°C freezer over night. The filter was poured in a falcon tube with 4 ml of acetone, crushed, and then extracted for 24 hours at 4°C. The suspension was shaken and filtrated through a glass microfiber binder free filter before measuring with the spectrophotometer the absorbance at 665 nm and 750 nm. Chlorophyll-a concentration [μg cm^{-2}] has been determined as [2] $\mathrm{Chl}\text{-}a=11.41(\mathrm{E_{665}}\text{-}\mathrm{E_{750}})(V_{\mathrm{acetone}}\cdot \mathrm{ratio_{Chl-a}})/A_{\mathrm{tile}}$ where $\mathrm{E_{665}}$ and $\mathrm{E_{750}}$ are absorbances at 665 nm and 750 nm respectively, $\mathrm{V_{acetone}}$ [ml] is the acetone volume for Chl-a filter, and $\mathrm{ratio_{Chl-a}}$ is the ratio between the total suspension volume (from whole scraped biofilm biomass) and the suspension volume used for the Chl-a filter.

Image analysis

Digital photos of sampled tiles (Figure 5A, B) during the grazed phase were analysed with a Matlab code to evaluate the grazed area portions on each tile. Single spots of grazed areas (i.e., white portions on the tiles) were determined visually on the original pictures, while the Matlab code identified all pixels having the same range of `[r g b]` values as the selected spot (chosen `[r g b]`, expressed as an integer in the range [0,255] $\pm$ 20), and reproduced the result on a new black (ungrazed) and white (grazed) tile image. A comparison between the original and the generated picture was carried out, in order to visually inspect the match between grazed areas. The evaluation of the white area on each tile, expressed as a percentage of the total area of the tile, was necessary to determine the normalized-ungrazed biofilm biomass during grazing activity. The normalized-ungrazed biofilm biomass is an estimate of the biofilm biomass that would have been expected in the absence of grazers. Therefore the normalized-ungrazed OM and Chl-a concentrations of biofilm biomass were derived from the measured values of OM or Chl-a during the grazed phase, referred to the ungrazed portion of the sampled tile by means of a simple proportion.

Autotrophic community composition of benthic biofilms as controlled by flow and light and potential effects on *Ecdyonurus* grazing rates

We tested if the effects of flow and light on grazing rates were mediated by changes in the autotrophic community composition of benthic biofilms (ACC) using a canonical correlative approach. ACC was determined by identification of algal cells in 8 representative samples collected at the onset of the grazing phase from all flow and light treatments. Biofilm samples were scraped from 5.8 cm^2 (i.e., a quarter of a ceramic tile) and stored in 3.6% formaldehyde. In Utermöhl counting chambers [3] algal cells were identified from a cell suspension from 1:100 up to 1:500 depending on the light treatment. For every sample at least 5 Utermöhl chambers were counted, adding up to at least 3,000 identified cells corresponding to 0.6-0.98 mm^2 of area covered with biofilm. Overall 68 algal taxa were microscopically differentiated at the genus level and counted.

First, ACC was analysed using non-metric multidimensional scaling (NMDS) based on a Bray-Curtis dissimilarity matrix computed from relative abundances [4]. The resulting ordination (Figure 4B) pointed to clear shifts in ACC due to flow stochasticity and across the light gradient. Flow-driven and light-driven shifts were comparable in magnitude and occurred along separate ordination axes indicating independent variation due to these two controls. The effects of flow and light on ACC were further tested by canonical analysis of principal coordinates (CAP) run on the Bray-Curtis dissimilarity matrix and using flow and light as additive constraints. Briefly, CAP [5] as done here consists of a translation of the semi-metric Bray-Curtis matrix into an Euclidean space by principal coordinate analysis (PCoA), followed by a redundancy analysis of the PCoA-axes using the flow and light treatments simultaneously as constraining variables. Significance of the whole CAP-model [5] can be tested by permutation and a pseudo-F value as test statistic; similarly significances of separate canonical axes can be tested by a forward or marginal method [6]. Even though our analysis lacked statistical power due to low sample size, the CAP-model was highly significant (pseudo-F=5.7, $P<0.001$). CAP further identified two canonical dimensions, along which ACC shifts as a response to flow stochasticity (first CAP axis, pseudo-F=7.82, $P<0.01$) and to the light gradient (second CAP axis, pseudo-F=3.52, $P<0.05$), which corroborates the NMDS-derived suggestion of ACC to respond differently and independently to flow stochasticity and the light gradient. Neither the flow-induced nor the light-induced shift of ACC was correlated with *Ecdyonurus* grazing rates (all $R<0.31$, all $P>0.46$). We then used CAP once more to test for an association of ACC with *Ecdyonurus* grazing rates. While this also necessarily identified one canonical dimension along which ACC shifts might be influencing grazing rates, the CAP model was not significant for OM grazing rates (pseudo-F=0.64, P=0.60) nor for Chl-a based grazing rates (pseudo-F=0.70, P=0.60) as separately considered constraints. Last, we investigated correlations among the canonical dimensions, i.e., the shifts of ACC driven by flow and light and those potentially associated with *Ecdyonurus* grazing (Figure S5 in

Supporting Information). Light- and flow-driven shifts were not correlated, and neither were light-driven and grazing-associated shifts. However, flow-driven shifts and grazing-associated shifts were significantly correlated (though suffering from a bimodal distribution). Taken together, these results and the analysis of grazing rates as dependent on flow and light (Figure 5C) suggest flow stochasticity as a common and strong control on both ACC and *Ecdyonurus* grazing. ACC is further (and almost equally strongly, Figure 4B) affected by light but not associated with grazing rates. This renders ACC an unlikely mediator of flow effects on *Ecdyonurus* grazing. Even though possible as ACC responds to flow independently of light, grazing would experience exclusive control by the ACC shifts between flow treatments, but not at all by the equally strong ACC shifts along the light gradient.

References

1. Lamberti G, Resh V (1985) Comparability of introduced tiles and natural substrates for sampling lotic bacteria, algae and macroinvertebrates. Freshwater Biology 15: 21-30.

2. Hauer F, Lamberti G (2007) Methods in Stream Ecology – Second Edition. Academic Press, London.

3. Utermöhl H (1958) Zur Vervollkommnung der quantitativen Phytoplankton-Methodik. Mitteilungen Internationale Vereiningung für Theoretische und Angewandte Limnologie 9: 1-38.

4. Legendre P, Legendre L (1998) Numerical Ecology – Second Edition. Elsevier Science B. V., Amsterdam, The Netherlands.

5. Anderson M, Willis T (2003) Canonical analysis of principal coordinates: A useful method of constrained ordination for ecology. Ecology 84: 511-525.

6. Legendre P, Oksanen J, ter Braak C (2011) Testing the significance of canonical axes in redundancy analysis. Methods in Ecology and Evolution 2: 269-277.

Table S1. Areal abundance of *Ecdyonurus* larvae in Austrian pre-alpine streams from field surveys (BOKU University, Vienna).

River	Individuals/m^2
OSB	280-380
Ybbs River	363
Mürz River	572
Triesting	142
Mayerhofer Bach	251

Table S2. Autotrophic Index (ratio of OM to Chl-*a*) for each discharge (S for stochastic, C for constant) and light treatment (mean ± SD).

% transmission	S	C	S+C
90%	381±68	373±82	377±73
65%	345±71	298±75	320±74
50%	256±45	292±46	273±48
27%	252±43	219±51	237±48
all light	307±79	300±84	304±81
